# Microsporidian *Nosema bombycis* secretes serine protease inhibitor to suppress host cell apoptosis *via* Caspase BmICE

**DOI:** 10.1371/journal.ppat.1012373

**Published:** 2025-01-07

**Authors:** Maoshuang Ran, Jialing Bao, Boning Li, Yulian Shi, Wenxin Yang, Xianzhi Meng, Jie Chen, Junhong Wei, Mengxian Long, Tian Li, Chunfeng Li, Guoqing Pan, Zeyang Zhou

**Affiliations:** 1 State Key Laboratory of Resource Insects, Southwest University, Chongqing, China; 2 Chongqing Key Laboratory of Microsporidia Infection and Control, Southwest University, Chongqing, China; 3 Key Laboratory of Conservation and Utilization of Pollinator Insect of the Upper Reaches of the Yangtze River (Co-Construction by Ministry and Province), Ministry of Agriculture and Rural Affairs, Chongqing Normal University, Chongqing, China; Chinese Academy of Sciences, CHINA

## Abstract

Microsporidia are a group of intracellular pathogens that actively manipulate host cell biological processes to facilitate their intracellular niche. Apoptosis is an important defense mechanism by which host cell control intracellular pathogens. Microsporidia modulating host cell apoptosis has been reported previously, however the molecular mechanism is not yet clear. In this report, we describe that the microsporidia *Nosema bombycis* inhibits apoptosis of *Bombyx mori* cells through a secreted protein NbSPN14, which is a serine protease inhibitor (Serpin). An immunofluorescent assay demonstrated that upon infection with *N*. *bombycis*, NbSPN14 was initially found in the *B*. *mori* cell cytoplasm and then became enriched in the host cell nucleus. Overexpression and RNA-interference (RNAi) of NbSPN14 in *B*. *mori’* embryo cell confirmed that NbSPN14 inhibited host cells apoptosis. Immunofluorescent and Co-IP assays verified the co-localization and interaction of NbSPN14 with the BmICE, the Caspase 3 homolog in *B*. *mori*. Knocking out of BmICE or mutating the BmICE-interacting P1 site of NbSPN14, eliminated the localization of NbSPN14 into the host nucleus and prevented the apoptosis-inhibiting effect of NbSPN14, which also proved that the interaction between BmICE and NbSPN14 occurred in host cytoplasm and the NbSPN14 translocation into host cell nucleus depends on BmICE. These data elucidate that *N*. *bombycis* secretory protein NbSPN14 inhibits host cell apoptosis by directly inhibiting the Caspase protease BmICE, which provides an important insight for understanding pathogen-host interactions and a potential therapeutic target for *N*. *bombycis* proliferation.

## Author summary

Microsporidia constitute a class of eukaryotic pathogens that exclusively reside within host cells. The species *Nosema bombycis* is the first microsporidia identified as the pathogen of silkworm Pébrine disease. In our research, we discovered how *N*. *bombycis* cleverly evades the host’s defenses. It has developed a strategy to survive inside host cells by manipulating apoptosis and disarming the host cell’s self-destruct mechanism. In this study, we discovered that the *N*. *bombycis* secretes a serine protease inhibitor named NbSPN14, which infiltrates the cytoplasm of the host cell. The NbSPN14 interacts with the executioner Caspase protease BmICE within the silkworm’s apoptotic pathway, effectively neutralizing its apoptotic activity and thus curbing the apoptosis of the host cells.

## Introduction

Microsporidia are a large group of single-celled, eukaryotic, obligate intracellular pathogens which infect both vertebrates and invertebrate hosts [1,2] The first microsporidia species was identified in 1857, which described as the *Nosema bombycis* [3], as the causative pathogen of Pébrine disease in silkworms [4]. Since then, more than 1700 species in 220 genera have been identified [5]. Microsporidia infections cause significant economic loss to animal husbandry and are threats to public health [6,7].

Apoptosis is a key feature of eukaryotic cells and is essential for the proper development of multicellular organisms [8]. Caspases are proteolytic enzymes that belong to the cysteine protease family and play a crucial role in apoptosis [9]. Caspase 3 is the primary executioner caspase that cleaves cellular substrates, including structural proteins and DNA repair enzymes, leading to cell apoptosis [10]. In addition, as a defense mechanism, studies have demonstrated that host cells undergo apoptosis to purge invading pathogens. However, some pathogens, such as viruses and bacteria manipulate apoptosis to aid their intracellular survival [11–14]. The phenomenon of microsporidia modulating host cell apoptosis has been reported previously [15–20]. As early as 1999, Scalon et al. found that *Nosema algerae* (now called *Anncaliia algerae*) infected human lung fibroblasts cells (HLF) and did not induce apoptosis, the survival time of infected cells *in vitro* increased by several days compared to uninfected cells [21]. Aguila et al. have reported that *Encephalitozoon cuniculi* infection could suppress the apoptosis of host cells. Upon further analysis, it was discovered that the nuclear translocation of p53 and the activation of Caspase 3 was markedly attenuated in Vero cells post-infection with *E*. *cuniculi* [20]. Similarly, both *Nosema apis* and *Nosema ceranae* reduce host cell apoptosis in bee epithelial cells [17,18,22,23]. *N*. *bombycis* infecting the ovarian cells of *Bombyx mori* (BmN cells) has been shown to inhibit the host cell apoptosis by downregulating the expression of genes associated with the mitochondrial apoptosis pathway [19]. However, now which effector of microsporidia inhibit the host cell apoptosis is unknown.

Serine protease inhibitors (Serpins) are a group of protease inhibitors that are found in almost all organisms [24]. Serpins form complexes with target proteases, thereby, regulating various biological processes such as blood homeostasis [25], inflammatory responses [26], and cell apoptosis [27,28]. Serpins from pathogens have been reported to manipulate the host immune and apoptotic processes of host cells by regulating the activity of host proteases, which is beneficial for pathogen evasion against immune of host and pathogen proliferation [29]. SPI-2 and CrmA are the most extensively studied poxvirus serine protease inhibitors, they are non-essential for virus replication, but are involved in multiple immunomodulatory events [30]. It has been reported that SPI-2 and CrmA inhibit apoptosis and host inflammatory responses [31]. SPI-2 and CrmA target Caspase 1 to protect virus-infected cells from TNF-mediated and Fas-mediated apoptosis as well as to prevent the proteolytic activation of interleukin-1β [32,33]. In the Microsporidia, a total of 53 serpins have been exclusively identified within the genus *Nosema*, and of these, 21 possess predicted signal peptides. Notably, nineteen serpin members have been identified in *N*. *bombycis*, eight serpins with predicted signal peptides. The phylogenetic analysis demonstrated that the *N*. *bombycis* serpins clustered with the poxvirus serpins [34,35]. In general, above clues clearly suggest that the serpin protein encoded by *N*. *bombycis* may be involved in inhibiting host cell apoptosis.

Herein, we report that *N*. *bombycis* secretes a serpin protein, NbSPN14 into host cell cytoplasm, where it directly interacts with silkworm homolog Caspase 3, BmICE, the key executing effector in the silkworm apoptosis pathway. Subsequently, NbSPN14 inhibits the BmICE activity to suppress the host cell apoptosis, which ultimately facilitate the intracellular proliferation of *N*. *bombycis*.

## Results

### *N*. *bombycis* infection inhibits *B*. *mori* cell apoptosis

**Fig 1:**
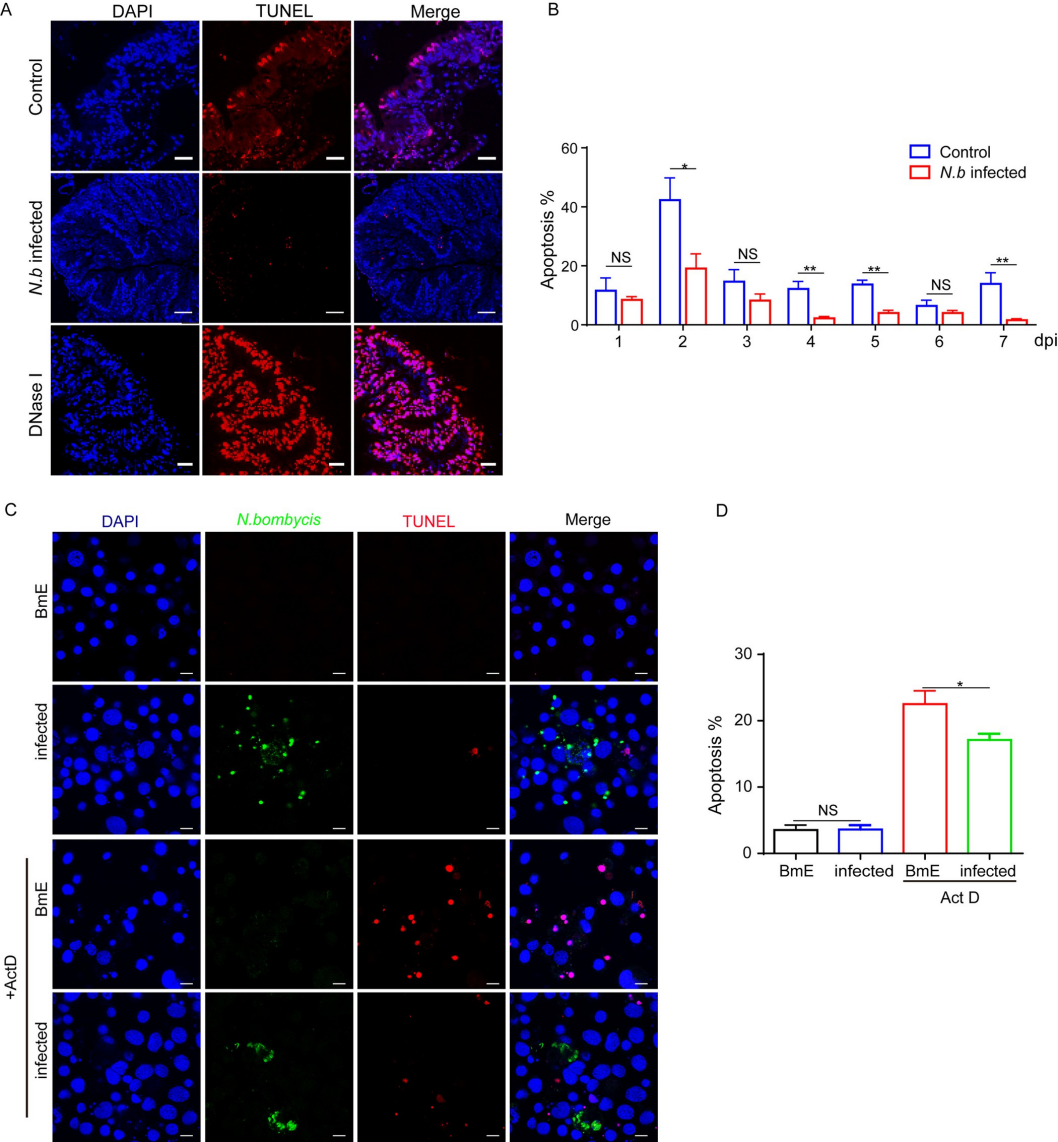
*N*. *bombycis* infection inhibits host cell apoptosis. A. TUNEL assay to detect apoptotic cells in transverse sections of infected and uninfected silkworm midgut, blue: nucleus, red: TUNEL positive signal, Control is silkworm raised under normal conditions, *N*. *b* infected means silkworms infected with *N*. *bombycis*, and the midgut cell treated with DNase I to establish a positive control, simulating DNA fragmentation associated with apoptosis. B. Quantitation of apoptosis in silkworm midgut cells 1 to 7 days after infection with *N*. *bombycis* (Ten randomly fields were used for statistical apoptosis ratio). C. TUNEL analysis of the host cell apoptosis with *N*. *bombycis* infection at 48 h, green: *N*. *bombycis*; red: TUNEL positive signal; scale bar, 10 μm; D. Quantitation of apoptosis of BmE cells infected with *N*. *bombycis* for 48 h (Six randomly fields were used for statistical apoptosis ratio).

The presence of *N*. *bombycis* within infected host midgut tissues and the cell line was verified using an immunofluorescent assay by Hochest33342 nuclear staining. There is a large number of pathogens present in the midgut cells of infected silkworms (S1A Fig). Terminal deoxynucleotidyl transferase dUTP nick end labeling (TUNEL) assay was then used to detect DNA fragmentation in host cells. Compared to un-infected and DNase I-treated positive controls, TUNEL signal in the midgut tissues of infected silkworms were significantly reduced (Fig 1A). The results from 1 to 7 days after infection were quantified by averaging the numbers of apoptotic cells ten observed fields under confocal microscopy. As shown in Fig 1B, the midgut cell apoptosis was significantly decreased after *N*. *bombycis* infection. In addition, the silkworm embryonic cell line, BmE was applied to confirm the above observations. The BmE cells were either un-infected, infected by *N*. *bombycis*, or further treated with the apoptosis inducer actinomycin D (Act D). The results demonstrated that there was no significant difference in apoptosis between *N*. *bombycis*-infected and un-infected groups (Fig 1C). However, when the two groups of cells were treated with actinomycin D, the apoptosis of *N*. *bombycis*-infected BmE cells was significantly lower than that of the uninfected control (Fig 1D). Then, we determined the Caspase 3 activity in *N*. *bombycis*-infected cells both before and after treatment with Act D. The findings revealed an elevation in Caspase 3 activity post-infection. However, upon Act D treatment, the Caspase 3 activity in the infected cells notably diminished compared to uninfected cells (S1B Fig). The above results indicated that *N*. *bombycis* infection inhibits host cell apoptosis.

### *N*. *bombycis* secretes NbSPN14 into its host cell

NbSPN14, as a member of the serpin family, is predicted to possess a secretion signal peptide (1–19 amino acids) [36]. AlphaFold analysis demonstrates that NbSPN14 has a typical Serpin domain and a reactive center loop (RCL) that "traps" target proteases. This feature suggests that NbSPN14 employs a canonical serpin mechanism to modulate biological processes (S2A Fig). The polyclonal antibody of NbSPN14 reacts with the corresponding antigenic band in *N*. *bombycis-*infected cell lysates thus confirmed the presence of this protein (S2B Fig). Transcripts of NbSPN14 could be detected after infection, with the highest transcript level at 48 h post-infection (S2C Fig).

**Fig 2:**
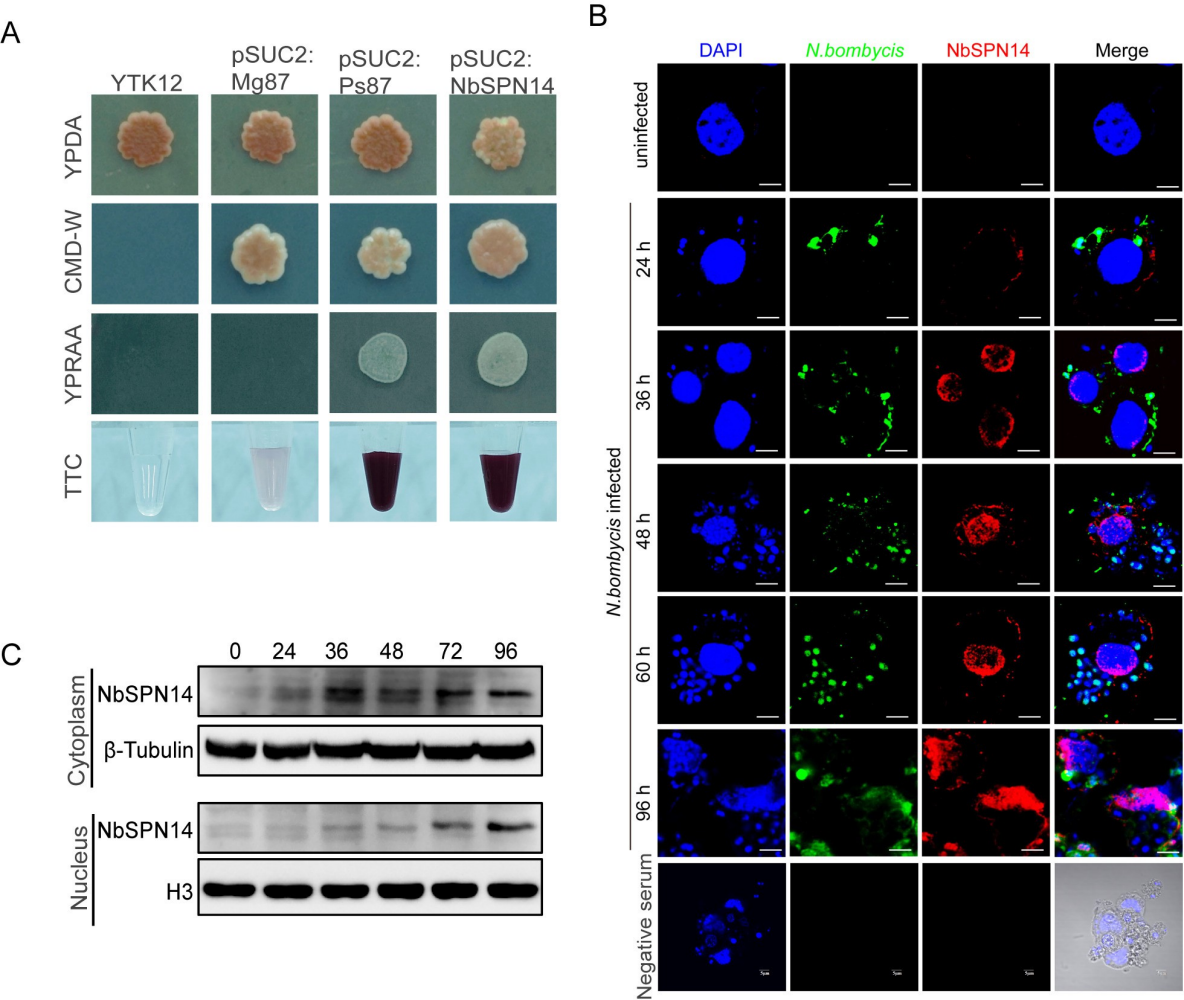
**N*. bombycis* secretes NbSPN14 into host cell nucleus. A. Yeast invertase secretion assay of the predicted signal peptide of NbSPN14. Yeast YTK12 strains carrying the NbSPN14 signal peptide fragments fused in frame to the invertase gene in the pSUC2 vector are able to grow in both the CMD-W media (with sucrose, yeast growth even in the absence of invertase secretion) and YPRAA media (with raffinose instead of sucrose, growth only when invertase is secreted), as well as reduce TTC to red formazan, indicating secretion of invertase. The controls include the untransformed YTK12 strain and YTK12 carrying the pSUC2 vector. B. Observations of the subcellular localization of NbSPN14 in infected BmE cells at 24, 36, 48, 60, 96 h post-infection by laser scanning confocal microscope. Nuclei are labeled with Hoechst 33342 (blue); *N*. *bombycis* labeled with rabbit anti-spore antibody and a goat anti-rabbit secondary antibody conjugated with Alexa Fluor 488 (green), NbSPN14 labeled with the anti-NbSPN14 mouse polyclonal antibody and a goat anti-mouse secondary antibody conjugated with Alexa Fluor 594 (red). C. Western blot analysis confirmed the distribution of NbSPN14 in the infected BmE cells at 24, 36, 48, 72, 96 h post-infection, with β-Tubulin serving as a cytoplasmic marker and H3 as a nuclear marker.

We confirmed that NbSPN14 has a functional secretory signal peptide by using Yeast signal sequence trap system (YSST), which allowed yeast growth on raffinose and TTC color change for the YTK12 yeast strain, while the negative control did not show these effects (Fig 2A). As shown in Fig 2B, while *N*. *bombycis* was labeled by the specific antibody against *N*. *bombycis* whole proteins, the NbSPN14 was identified within host cells. Further analysis demonstrated that at 36 h after infection, NbSPN14 was mostly localized in the host cytoplasm, at 48–60 h post-infection, NbSPN14 had partially translocated into the host cell nucleus; and at 96 hours after infection (when the pathogenic load is very high) most of the NbSPN14 was translocated into the host cell nucleus. Western blot analysis confirmed that the distribution of NbSPN14 in the nucleus of host cells increased with infection time, with β-Tubulin serving as a cytoplasmic marker and H3 as a nuclear marker. (Fig 2C) The transient expression of V5-tagged NbSPN14 in BmE cells also demonstrated that NbSPN14 could localize to the cytoplasm and nucleus of host cells (S2D Fig). These findings confirm that NbSPN14 is a secreted protein, and the localization of NbSPN14 to the host cell nucleus suggests that NbSPN14 may interact with a host cell protein during *N*. *bombycis* proliferation.

### NbSPN14 inhibits host cell apoptosis

**Fig 3:**
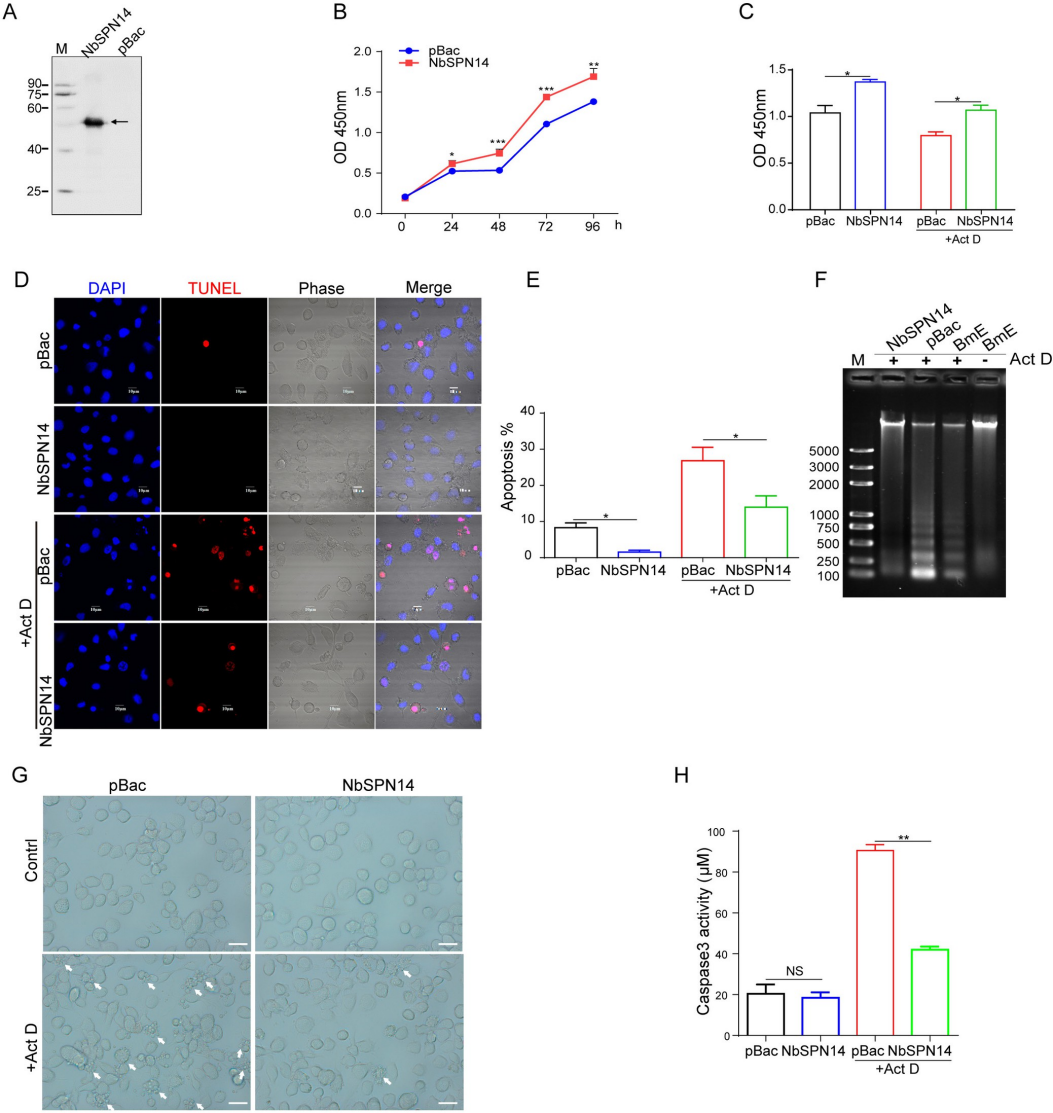
Inhibition of host cell apoptosis by overexpression of NbSPN14 in BmE cell line. A. Western blotting was used to verify the expression of NbSPN14 in the transgenic cells. B. CCK-8 analysis of the cell proliferation after continuous culture of NbSPN14 transgenic cells at 0–96 h, and the x-coordinate was the continuous culture time. C. CCK-8 analysis of the NbSPN14 transgenic cells treated with Act D at 48 h. D. TUNEL analysis the apoptosis of BmE-NbSPN14 transgenic cell line, red: TUNEL positive signal means the cell in a state of apoptosis. E. Quantification of the NbSPN14 transgenic cells apoptosis rate induced by Act D, based on TUNEL results (n = 20 filed each group). F. DNA ladder analysis was used to detect the apoptosis of NbSPN14 transgenic cells after treatment with Act D, the transgenic empty vector pBac and BmE cells were used as controls. G. Morphological observation of NbSPN14 transgenic cells after induction of apoptosis by Act D. Arrows indicate apoptotic cells, which are shrinking and bubbling to form many apoptotic vesicles. H. Caspase 3 activity analysis of NbSPN14 transgenic cells.

In order to study NbSPN14 function, NbSPN14 was expressed in cells to mimic the NbSPN14 secretion of pathogens into host cells. The transgenic cell line expressing NbSPN14 without the signal peptide sequence was successfully constructed (Fig 3A). CCK8 assays demonstrated that transgenic BmE cells expressing NbSPN14 possessed significantly higher cell viability compared to the pBac empty vector cell line (Fig 3B). Next, both groups of cells were treated with Act D, it was found that the proliferation activity of NbSPN14 transgenic cells were significantly higher than the control (Fig 3C). TUNEL assay results showed that Act D could strongly induce apoptosis of pBac cells, and the cell number of apoptosis increased significantly after treatment with Act D; However, the apoptosis rate of the transgenic cell line expressed NbSPN14 was significantly lower than that of pBac cell (Fig 3D–3E). Apoptosis can be visualized as a ladder pattern of 180–200 bp in standard agarose gel electrophoresis due to DNA cleavage by the activation of a nuclear endonuclease. The result showed the formation of the DNA ladder in gel electrophoresis by induction of apoptosis in NbSPN14 transgenic cells is much weaker than that of the control cell (Fig 3F). Similarly, a larger number of apoptotic cells with the apoptotic body can be clearly observed in the control group, compared with minor apoptotic cells in the NbSPN14 transgenic cells (Fig 3G). Annexin V staining is a common method for detecting apoptosis. By using Annexin V-mCherry to stain the live NbSPN14 transgenic cells, we observed that after treatment with Act D, the number of cells labeled by Annexin V-mCherry was significantly lower than that of the pBac cells (S3A and S3B Fig). What’s more, Caspase 3 activity is an important indicator of apoptosis, as shown in Fig 3H, compared with control, the activity of Caspase 3 in the transgenic cell line expressing NbSPN14 was decreased significantly after Act D treatment, indicating that NbSPN14 play an important role in inhibiting host cell apoptosis.

**Fig 4:**
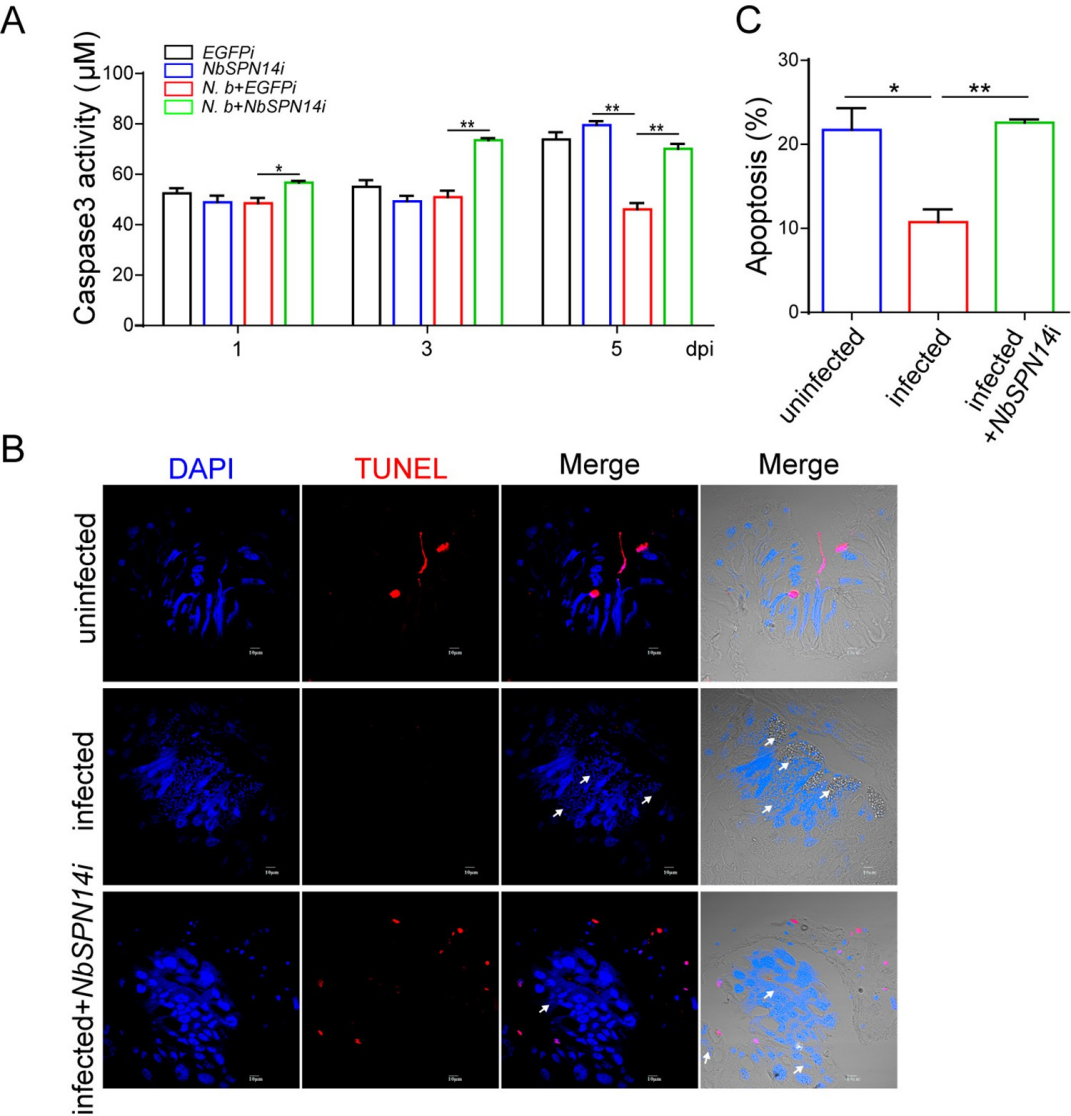
Host cell apoptosis increased after down-regulation of NbSPN14 expression by RNAi interference. A. Analysis of Caspase 3 activity in cells with NbSPN14-RNAi was significantly lower than the control group. B. The apoptosis of midgut cells of silkworms infected by *N*. *bombycis* were analyzed by TUNEL, the apoptotic signal of midgut cells increased significantly after NbSPN14 interference. C. Quantitation of apoptosis of midgut cells infected with *N*. *bombycis* for 4 d after NbSPN14 interference (Five randomly fields were used for statistical apoptosis ratio).

To further confirm the key function of NbSPN14 in inhibiting host cell apoptosis, we down-regulated the expression of NbSPN14 by transfecting the double-stranded RNA interference fragment targeting NbSPN14 gene into infected cells. The expression of NbSPN14 was detected by quantitative PCR and Western blotting, the results showed that the expression of NbSPN14 was down-regulated compared with the control group transfected with EGFP double-stranded dsRNA interference fragments (S4A–S4C Fig). The results of Caspase 3 activity assay showed that the infection of *N*. *bombycis* could inhibit host cells’ Caspase 3 activity, down-regulating the expression of NbSPN14 led to the increase of Caspase 3 activity of infected cells (Fig 4A). We utilized RNAi to block the expression of NbSPN14 in *N*. *bombycis* infected silkworm larvae. NbSPN14-dsRNA and the control EGFP-dsRNA were injected into the silkworms respectively after *N*. *bombycis* infection. The expression of NbSPN14 of the midgut determined was drawn from 3 dpi to 4 dpi (S4D–S4F Fig), and the results showed that NbSPN14 expression was successfully knocked down. TUNEL analysis result of midgut cell apoptosis after NbSPN14 interference was shown in Fig 4B. Compared with the uninfected group, the infected group midgut cells with the positive signals of TUNEL were significantly reduced. The apoptosis was significantly increased in response to the interference of NbSPN14 expression in the silkworm midgut compared with the infected group (Fig 4C). These results confirmed that NbSPN14 is an important effector in *N*. *bombycis* inhibiting host cell apoptosis.

### NbSPN14 inhibiting apoptosis increases *N*. *bombycis* proliferation

**Fig 5:**
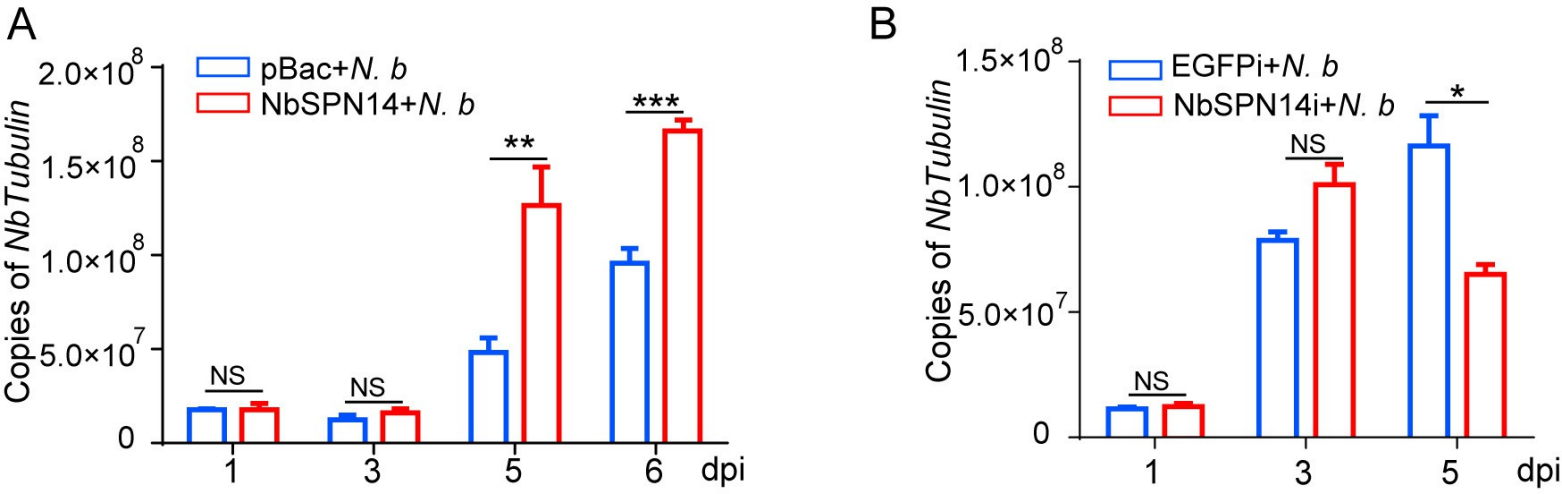
NbSPN14 can increase the proliferation of *N*. *bombycis* by inhibiting cell apoptosis. A. The *β-tubulin* copies level in BmE-NbSPN14 transgenic cells was higher than that of empty Vector control cells in *N*. *bombycis* infection. B. The *β-tubulin* level in infected BmE cells knock-down NbSPN14.

To explore whether NbSPN14 inhibiting host cell apoptosis promotes the proliferation of pathogens in host cells, the proliferation of *N*. *bombycis* in host cells was analyzed following *N*. *bombycis* infection in NbSPN14 transgenic cell line and the infected cells with NbSPN14 interference, respectively. The copy number of *Nb β-tubulin* was used to the represent the pathogen load of the two groups. As shown in Fig 5A, the *Nb β-tubulin* copies in NbSPN14 transgenic cells were significantly higher than those in empty control cells at 5- and 6 dpi. However, the pathogen load in host cells was significantly reduced after NbSPN14 expression was down-regulated by RNA interference in infected cells at 5dpi (Fig 5B). The results showed that NbSPN14 inhibiting host cell apoptosis was important for *N*. *bombycis* intracellular proliferation.

### BmICE is the target enzyme of NbSPN14

**Fig 6:**
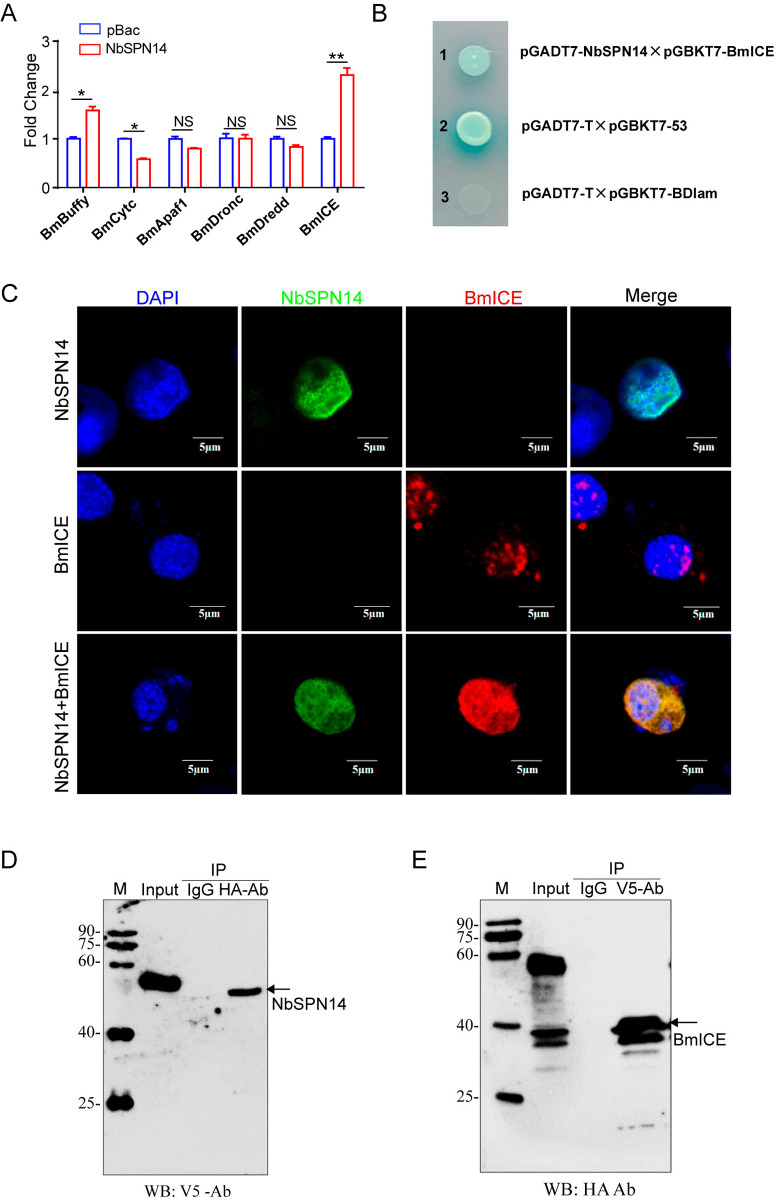
BmICE is the target protease inhibited by NbSPN14. A. Quantitative PCR analysis was used to analyze the transcription of *BmBuffy*, *BmCytc*, *BmApaf1*, *BmDronc*, *BmDredd*, and *BmICE* genes related to the apoptosis pathway in NbSPN14 transgenic cells. B. Yeast two-hybrid assay to verify the interaction between NbSPN14 and BmICE. Interaction of pGADT7-NbSPN14 with pGBKT7-BmICE was screened by SD/-Ade/-His/-Leu/-Trp/X-α-gal/AbA medium. C. Co-localization assay of NbSPN14-V5 and BmICE-HA co-expression in BmE cells. Nuclei were stained by DAPI, NbSPN14 was detected using a V5 mouse monoclonal antibody and an Alexa-488 secondary antibody, BmICE was detected using an HA rabbit monoclonal antibody and an Alexa-594 secondary antibody. D. Lysates from cells co-expressing protein NbSPN14-V5 and protein BmICE-HA were subjected to Co-IP using an anti-HA antibody, followed by Western blot detection with an anti-V5 antibody. E. Lysates from cells co-expressing protein NbSPN14-V5 and protein BmICE-HA were subjected to Co-IP using an anti-V5 antibody, followed by Western blot detection with an anti-HA antibody. Input: samples of co-expressed cells, IgG: negative control.

To investigate the target protein and the mechanism by which NbSPN14 inhibits the host cell apoptosis pathway, we analyzed Caspase 3 activity in cells following treatment with Ultraviolet light, Act D, and Staurosporine (STS). The results showed that the Caspase 3 activity of NbSPN14 transgenic cells after treatment with apoptosis inducers was significantly lower than that of the control group (S5A Fig). NbSPN14 can inhibit apoptosis triggered by a variety of apoptotic agents, suggesting that its targeted protein plays a pivotal role in the apoptotic pathway. Then, we analyzed the transcription of genes related to the apoptosis pathway in NbSPN14 transgenic cells, and found that the pro-apoptotic gene *BmICE* was significantly up-regulated. In contrast, while the upstream apoptosis-related genes *BmDronc* and *BmDredd* showed no significant changes (Fig 6A). Transcriptional analysis revealed a significant increase in BmICE levels; however, its activity was suppressed, which could be attributed to a rise in compensatory transcription. To ascertain whether NbSPN14 suppresses Caspase 3 activity by targeting upstream Caspase 9, we conducted enzyme activity assays. The results, depicted in S5C Fig, demonstrated that NbSPN14 is capable of inhibiting Caspase 3 activity instead of Caspase 9. Collectively, these findings imply that NbSPN14 may directly inhibit Caspase 3 activity.

BmICE, the silkworm homolog of Caspase 3, behaving as the key executing effector in silkworm apoptosis pathway, is composed of the P10 and P20 subunits and includes the conserved pentapeptide motif QACRG [37] (S5B Fig). It was predicted that the NbSPN14 P1 site at position 348th was aspartic acid D, which could be recognized by Caspase. Furthermore, we discovered that the amino acid located at the P1 site of NbSPN14 is identical to the amino acid found at the P1 site of CrmA, a serpin protein encoded by the Cowpox virus [33]. Notably, CrmA is known to effectively hinders host cell apoptosis by suppressing Caspase activity [38,39]. These finding hints that NbSPN14 could possibly employ a similar mechanism to achieve inhibition of host cell apoptosis.

Combined all the information above, we presumed that the candidate of the NbSPN14-inhibiting target protein was BmICE. To confirm the hypothesis, we utilized the yeast two-hybrid system, revealing a clear interaction between NbSPN14 and BmICE (Fig 6B). At the same time, we co-expressed NbSPN14 and BmICE in BmE cells, and found that they were co-localized in the host cytoplasm and nucleus, indicating that there may be an interaction between NbSPN14 and BmICE (Fig 6C). Co-IP experiments confirmed the interaction between V5-tagged NbSPN14 and HA-tagged BmICE in BmE cells, as evidenced by the detection of NbSPN14 with a V5 antibody following immunoprecipitation with a HA antibody, with reverse verification shown in Fig 6D and 6E. The above results indicate that the NbSPN14 could interact with BmICE directly.

**Fig 7:**
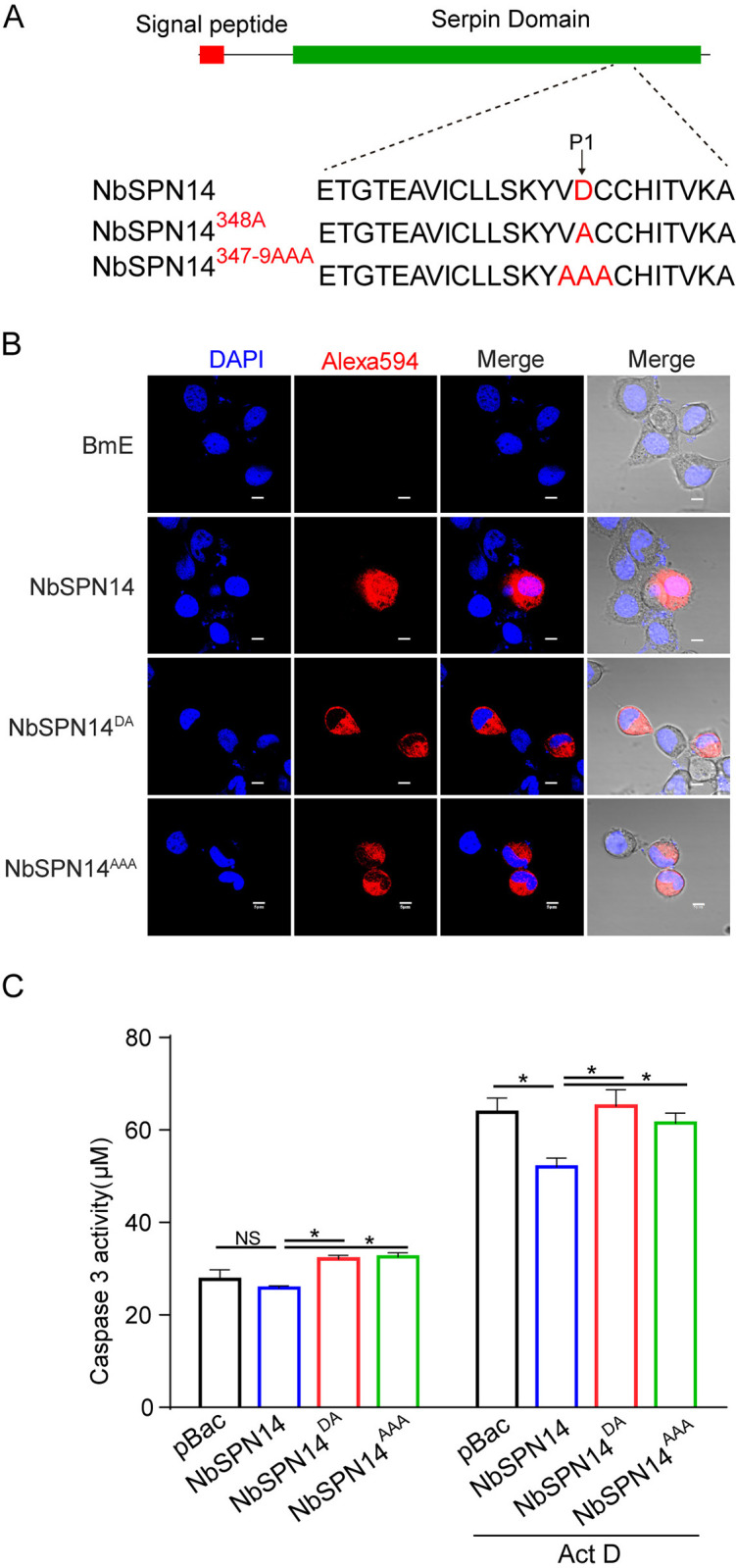
NbSPN14 P1 mutants are unable to enter the nucleus and inhibit apoptosis. A. NbSPN14 RCL sequence and P1 mutant schematic diagram. B. NbSPN14 and its mutant transient expression vector psL1180 [IE-NbSPN14mut-V5-SV40] were transfected into BmE cells for 48 hours to analyze the subcellular localization by immunofluorescence. The primary antibody uses the V5-tagged mouse monoclonal antibody, the secondary antibody was the Goat anti-Mouse IgG with Alexa Fluor 594. C. Caspase3 activity analysis of NbSPN14 and NbSPN14 mutation transgenic cells with Act D treated 12 h.

The amino acid residues of the P1 site in the RCL region of serpin protein play an important role in inhibiting target protease. Through site-directed mutagenesis, the 348th amino acid D of NbSPN14 was mutated to alanine A as NbSPN14^348A^, and the 347–349 amino acids were mutated into alanine A as NbSPN14^347-9AAA^, respectively (Fig 7A). Subcellular localization was analyzed after transient expression of NbSPN14 mutants in BmE cells. As shown in Fig 7B, NbSPN14 mutant (including NbSPN14^348A^, NbSPN14^347-9AAA^) only localized in cytoplasm, could not enter the host cell nucleus. After constructing the cell line expressing NbSPN14 mutant protein, then we evaluated the function of the NbSPN14 mutant to inhibit host cell apoptosis (Fig 7C). The results showed that the Caspase 3 activity of mutated NbSPN14 transgenic cells was higher than that of wild-type NbSPN14 transgenic cells, which indicates that the P1 site amino acid residue D played a decisive role in NbSPN14 inhibiting the BmICE Caspase activity.

### BmICE plays an important role in silkworm apoptosis

**Fig 8:**
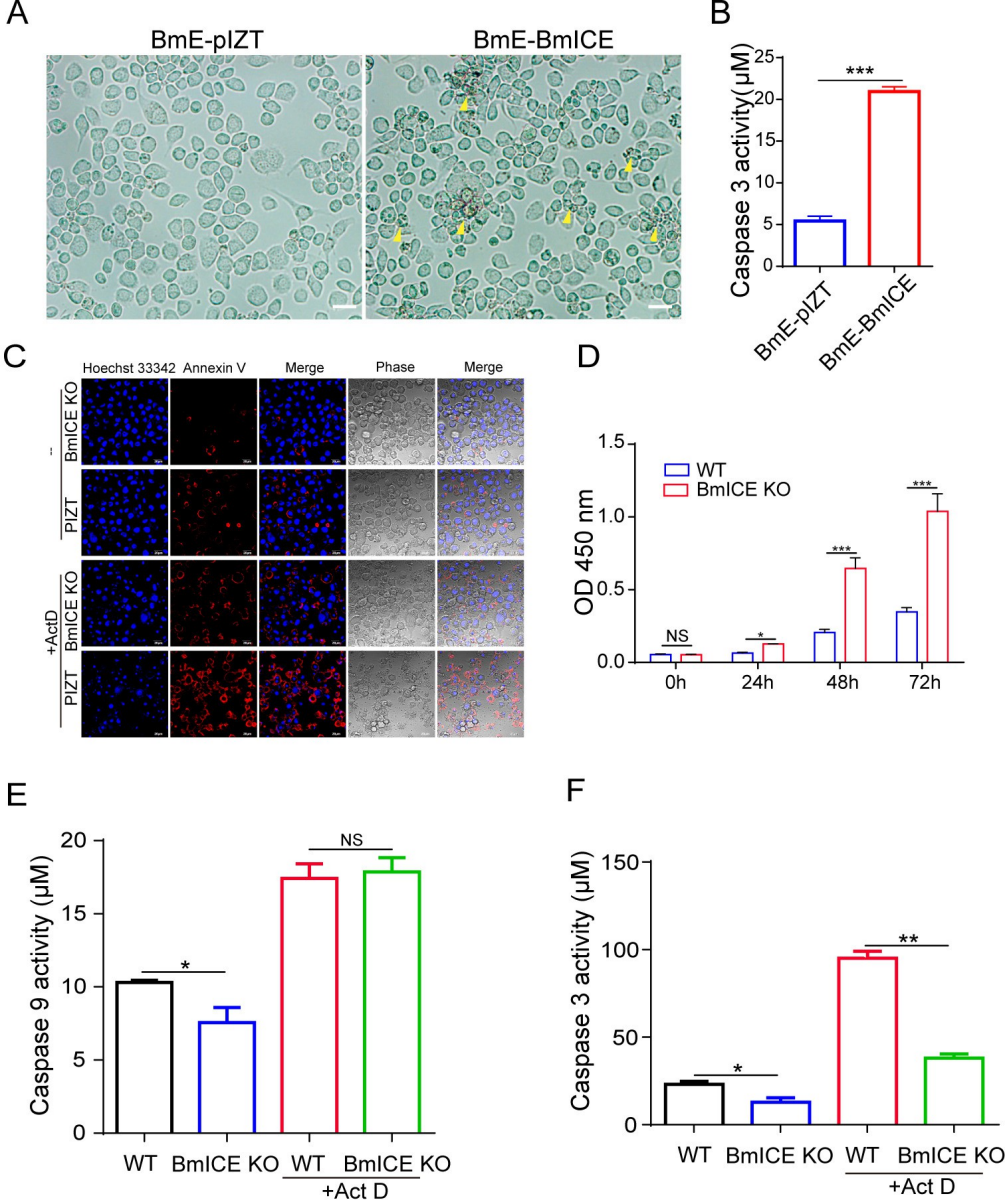
BmICE plays an important role in silkworm apoptosis. A. Observation of the morphology of BmICE overexpressed cells, yellow triangles heads indicate shrinking, foaming cells undergoing apoptosis. B: Caspase 3 activity of BmICE overexpressed cells were significantly higher than that of control cells with empty vector. C. The assessment of apoptosis in BmICE knockout cells treated with Act D was conducted using Annexin V-mCherry live cell staining analysis. The apoptosis of BmICE knockout cells was significantly less than that of the control group after treatment with Act D. D. CCK-8 analysis of the cell proliferation after continuous culture of BmICE knock out cells, and the x-coordinate was the continuous culture time. E. Analysis of Caspase 9 activity of BmICE knockout cells after Act D treatment showed no significant difference compared with the control group. F: Caspase3 activity of BmICE knockout cells after Act D treatment was significantly lower than that of control cells.

To ascertain the role of BmICE in the apoptosis pathway of silkworm cells, we constructed a BmICE-overexpressing cell line and monitored the growth status of the cells. The results indicated that the growth index of BmICE-overexpressing cells was inferior to that of the control group, with an increased number of cells exhibiting apoptotic morphology and a significant rise in Caspase 3 activity (Fig 8A and 8B). Immunofluorescence assay (IFA) analysis revealed that BmICE, when overexpressed in cells, is localized to both the cytoplasm and the nucleus. Notably, during periods when cells display apoptotic features, including nuclear condensation, there is a marked accumulation of BmICE within the nucleus (S6A Fig).

To further validate the function of BmICE in cell apoptosis, we used the CRISPR/Cas9 system to knock out the BmICE gene through using double gRNA guidance to cleave the fourth and fifth exons of the BmICE gene and inserting the GFP/Zeocin report/screened gene into its locus by homologous recombination. Quantitative PCR and Western blot results showed that BmICE was successfully knocked out (S6B and S6C Fig). Quantitative PCR results indicated that knocking out BmICE had no significant effect on the upstream apoptosis pathway (S6D Fig). After treatment with Act D, there was a significant upregulation of pro-apoptotic genes, with the exception of *BmICE*, indicating that the apoptotic pathway was been triggered (S6E Fig). However, in BmICE-knockout cells, the apoptosis induced by Act D treatment was effectively blocked. Through annexin V live cell staining to label apoptotic cells, it was found that BmICE knockout cells had fewer apoptotic cells than the control pIZT cells. And this difference became more pronounced after the induction of apoptosis with actinomycin D (Act D) treatment (Fig 8C). The proliferative activity of these cells was evaluated using the CCK-8 assay. Results showed that at 24-, 48-, and 72-hours post-cell seeding, the cell viability of BmICE knockout cells was significantly higher than that of the empty vector control group (Fig 8D). Then, the Caspase 3 and Caspase 9 activities in BmICE knockout cells were detected and the results showed that the Caspase 3 activity of the knockout cells was significantly lower than that of the control group, there was no significant difference in Caspase 9 activity between the BmICE knockout cells and the control group (Fig 8E and 8F). The results indicate that BmICE plays a crucial role in the apoptosis pathway of silkworm cells.

### NbSPN14 entering the host cell nucleus depends on BmICE

**Fig 9:**
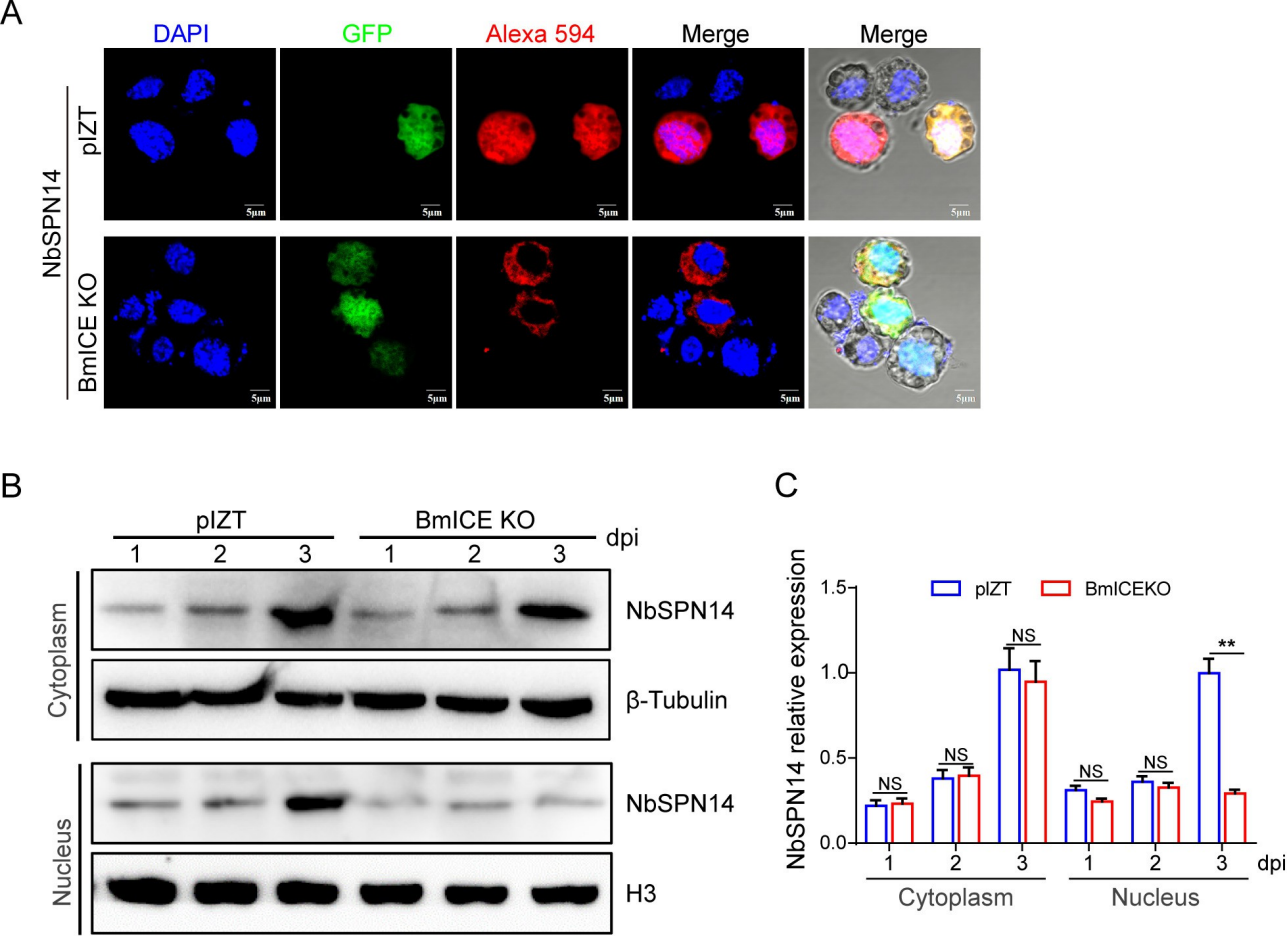
NbSPN14 entering the host cell nucleus depends on BmICE. A. Localization of transient expression of NbSPN14 in *BmICE* knockout cells. The recombinant NbSPN14 vector was transfected into *BmICE* knockout cells, and the location of recombinant protein NbSPN14-V5 in the cells was analyzed by V5 mouse monoclonal antibody with Alexa-594 conjugated secondary antibody. The results showed that NbSPN14 was not located in the host nucleus after BmICE knockout. B. Western blot analysis confirmed the distribution of NbSPN14 in the infected BmICE knock out cells at 1, 2, 3days post-infection, with β-Tubulin serving as a cytoplasmic marker and Histone H3 as a nuclear marker. C. Quantitative analysis was conducted on the distribution of NbSPN14 in the cytoplasm and nucleus of BmICE knockout cells. The NbSPN14 relative expression level is the ratio of the gray level of the NbSPN14 band to that of the internal reference β-tubulin band.

In infected BmE cells and the NbSPN14 transgenic cell line, we observed that NbSPN14 is localized to the cell cytoplasm and subsequently translocate into the cell nucleus. However, subcellular localization prediction analysis showed that NbSPN14 has no nuclear localization motif, while BmICE can enter the nucleus [40]. We assume that NbSPN14 enters the nucleus after interacting with the target protein BmICE in the host cytoplasm. To confirm our hypothesis, the transient expression vector of NbSPN14 was transfected into BmICE knockout cells, and the localization of NbSPN14 in BmICE knockout cells was analyzed by IFA (Fig 9A). Through western blot analysis of cytoplasmic and nuclear fractionation experiments, we examined the distribution of NbSPN14 in host cells at 1-, 2-, and 3-days post-infection with *N*. *bombycis* in BmICE knockout cells. The results showed that NbSPN14 accumulates in the cytoplasm of host cells over time, and the amount in the nucleus of BmICE knockout cells was significantly lower than that in the control group at 3 days post-infection (Fig 9B and 9C). The results showed that the localization signal of NbSPN14 was distributed only in the cytoplasm, but not in nucleus in BmICE-knockout cells, while NbSPN14 could be localized throughout the entire cell including the nucleus in control cells. The results indicated that NbSPN14 relied on the interaction with BmICE in the cytoplasm to enter the nucleus as NbSPN14-BmICE complex.

## Discussion

**Fig 10:**
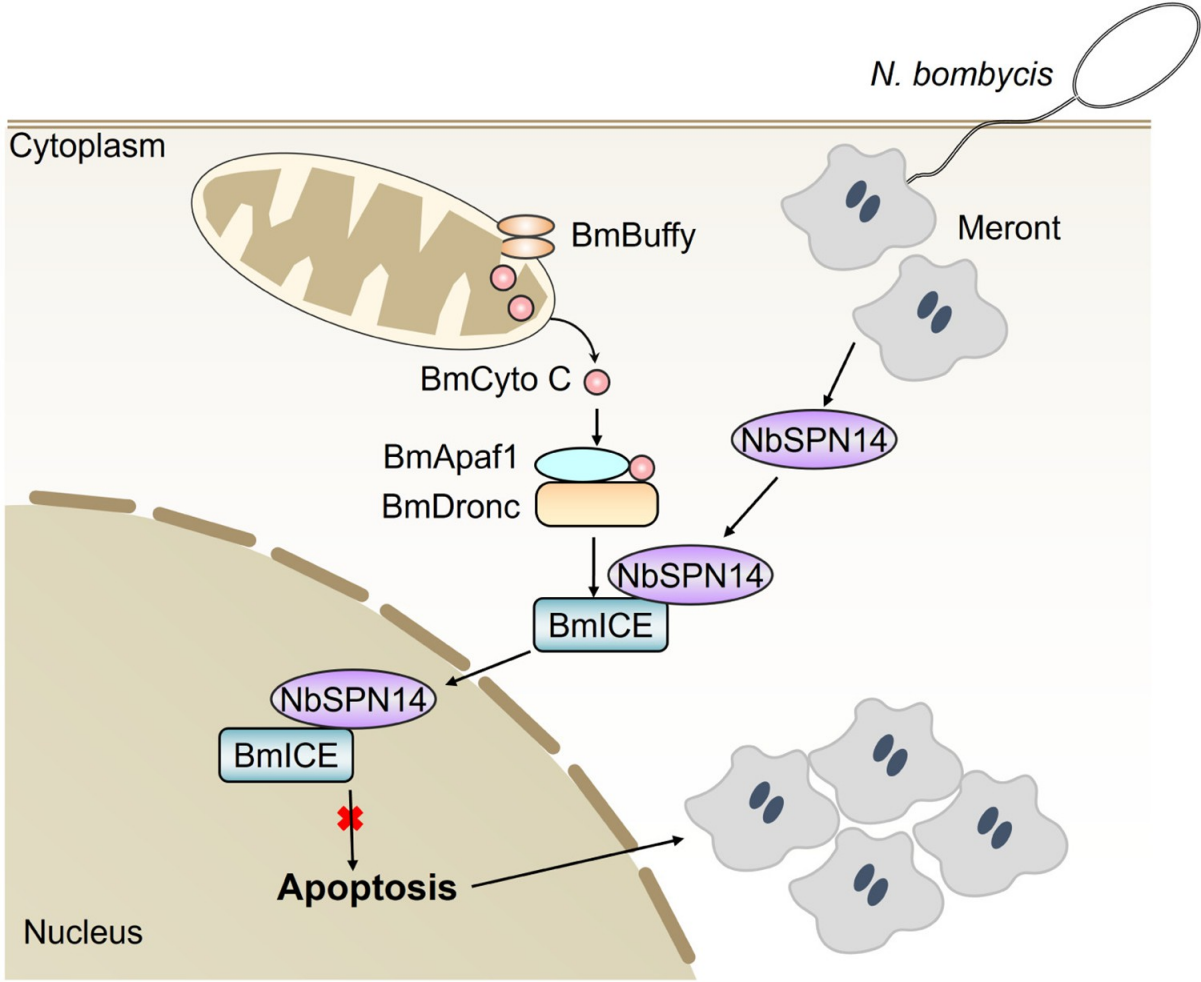
The schematic diagram of *N*. *bombycis* NbSPN14 inhibiting host cell apoptosis. *N*. *bombycis* infects the host cell through the polar tube injection and then the sporoplasm enters become the meront that is the fission and proliferation stage. NbSPN14 is secreted into the cytoplasm of host cells by *N*. *bombycis* meront. Then, NbSPN14 binds to and deactivated BmICE in the cytoplasm and subsequently translocated into the nucleus. NbSPN14 inhibits the BmICE activity, blocks apoptosis, and increases time and space for the *N*. *bombycis* proliferation.

As a group of intracellular eukaryotic parasites, it was reported that microsporidian infection could inhibit host cell apoptosis [36–41]. However, it was unclear which effector was secreted into host cells to inhibit apoptosis. In this study, we demonstrated that *N*. *bombycis* secretes NbSPN14 into the host cell and inhibits the activity of the key apoptotic effector Caspase enzyme BmICE, thereby inhibiting host cell apoptosis. NbSPN14 binds to the activated BmICE in the cytoplasm and inhibits the Caspase activity of BmICE; then the BmICE-Nbserpin14 complex enters the host cell nucleus. However, BmICE has been deactivated, losing its effector function of hydrolyzing cell components and thereby promotes pathogen proliferation in the infected host cell (Fig 10).

Phylogeny analysis indicates that *N*. *bombycis* serpin genes are clustered with the poxvirus serpin genes [35]. The P1 site amino acid residue of the NbSPN14 reactive center loop was Aspartic acid, which is consistent with that of the Cowpox-Virus serpin, CrmA. It has been reported that CrmA inhibits apoptosis in a variety of cells [31]. Moreover, CrmA also inhibits the activity of Caspase protease, interleukin-1 beta-converting enzyme (ICE) in *Caenorhabditis elegans* [41]. Thus, we speculated that the target protein of NbSPN14 was the *B*. *mori* homolog BmICE. The protein-protein interaction results confirmed that the target protein inhibited by NbSPN14 was BmICE. Although *N*. *bombycis* and poxvirus are far apart in the phylogenic tree of species, the molecular mechanism that *N*. *bombycis* secreting NbSPN14 to inhibit host cell apoptosis is similar to that of serpin in poxvirus [42].

Microsporidia, including *E*. *cuniculi*, *Vittaforma corneae*, *Nosema apis* and *Nosema ceranae*, their infection can inhibit host cell apoptosis [18,20,43]. However, the molecular mechanisms of inhibition of host cell apoptosis may not be identical in these microsporidia. The previous research results showed that microsporidia infection could inhibit host cell apoptosis through the typical Caspase pathway. The expression patterns of apoptosis-related genes were distinct after *E*. *cuniculi* and *V*. *corneae* infection of human macrophages THP-1, respectively; however, the Cysteine-aspartate protease (Caspase 3) activity was inhibited after THP-1 macrophages infected by both *E*. *cuniculi* and *V*. *corneae* [43]. Similarly, *E*. *cuniculi* infection prevented Caspase 3 cleavage in Vero cells [20]. Immunocytochemistry results demonstrated the depletion of Caspase 3 in the ventricular epithelial cells of honeybees infected with *N*. *ceranae* [22]. In this study, we confirmed that the secretion of NbSPN14 by *N*. *bombycis* inhibits Caspase activity and inhibits host cell apoptosis. However, in the microsporidia, now only the *Nosema* genus has serpin family genes annotated, other microsporidia without serpin may inhibit host cell apoptosis through other effectors or different molecular mechanisms. Apoptosis Inhibitory Proteins (IAPs) are a highly conserved family of anti-apoptotic factors, which act directly on the Caspase family and inhibit their activity [44]. IAP genes have been identified in a variety of microsporidia [45], so further study should focus on their functions involved in the regulation of host cell apoptosis. In addition, whether Serpins of other species of the *Nosema* genus are involved in inhibiting host cell apoptosis needs to be verified.

NbSPN14 is dependent on BmICE for entry into the host cell nucleus. NbSPN14 has a predicted secreted signal peptide but no nuclear localization signal (NLS). In the early stages of *N*. *bombycis* infection, NbSPN14 is secreted into the host cytoplasm, and then translocated to the host nucleus as the infection progresses (Fig 2). In the current study we have shown that NbSPN14 localizes to the host cell cytoplasm and interacts with BmICE. The interaction leads to the loss of Caspase activity and the inhibition of host cell apoptosis. A previous study has shown that the Caspase 3 homolog BmICE acts as an effector factor in regulating apoptosis [37,46]. Caspase 3 is predominantly localized in the cytoplasm in the form of pro-enzyme [40]. During the process of apoptosis, activated Caspase 3 is translocated into the nucleus to cleave its nuclear substrates, resulting in characteristic apoptotic nuclear changes such as DNA fragmentation, chromatin agglutination, and nuclear disruption [47,48]. We demonstrated that NbSPN14 binds to BmICE in the host cytoplasm and then was translocated to the host cell nucleus. We expressed NbSPN14 in BmICE knockout cells and found that NbSPN14 could not be translocated into the host cell nucleus, confirming that NbSPN14 inhibits BmICE activity in the cytoplasm and then be translocated into the host cell nucleus along with BmICE.

NbSPN14 inhibits host cell apoptosis, which increases pathogen proliferation. Intracellular infection depends on the survival of host cells for pathogen proliferation [49]. Inhibition of host cell apoptosis by pathogenic secreted effectors is a common strategy for the survival of intracellular pathogens [50]. Herein, we demonstrated that NbSPN14 is a key effector molecule in host cell apoptosis inhibition, which is essential for robust *N*. *bombycis* proliferation. It raises the possibility that therapeutic compounds targeting this pathway or genetic manipulation of the host could protect silkworms from infection with *N*. *bombycis*. Previous studies have shown that the transgenic expression of monoclonal antibody single-chain variable fragments (scFvs) targeting *N*. *bombycis* protein in silkworm or host cell lines reduced *N*. *bombycis* infection and proliferation [51–53]. As host cell apoptosis promotes the clearing of infected cells and reduces the load of *N*. *bombycis*, we can express monoclonal antibody scFv against NbSPN14 in silkworm to block the *N*. *bombycis* serpin inhibiting host cell apoptosis; as a result, the resistance of the host silkworm to *N*. *bombycis* will enhance. In addition, serpin usually contains a conformation relaxed reactive center loop (RCL) that is cut between P1 and P1’ residues by a target protease [54,55]. The RCL P1 site amino acid residues are critical for serpin function and specificity [56]. After the reaction between the protease and serpin, the protease is trapped in a covalent complex with serpin [57]. Consequently the RCL is rapidly incorporated as a new central β-strand into the serpin A β-sheet [58]. It has been reported that specific RCL-derived peptides could mimic the RCL insertion strand within serpin domain 4A [59–62], and cause the serpin function loss [63]. Therefore, over-expression or assembling the NbSPN14-derived peptides within host cells would be a feasible and novel strategy to combat with the inhibition effects of microsporidia on host apoptosis and ultimately bolster the silkworms’ resistance against pebrine diseases.

## Materials and methods

### Silkworm rearing and cell culture

The silkworm strain Dazao was obtained from the Gene Resource Library of Domesticated Silkworm (Southwest University, Chongqing, China). Silkworms were reared with fresh mulberry leaves at a temperature of 25°C and relative humidity of 70%. Silkworm cell lines were maintained at 28°C in Grace’s medium (Thermo Fisher Scientific) supplemented with 10% (V/V) fetal bovine serum (FBS) (Thermo Fisher Scientific) and 1% (V/V) penicillin-streptomycin (Beyotime) [64].

### *N*. *bombycis* infections *in vitro* and *in vivo*

Mature *N*. *bombycis* spores were isolated from infected silkworm pupae and purified by Percoll density gradient centrifugation (21,000g, 40 min) [65]. Purified *N*. *bombycis* mature spores were treated with 0.1 M KOH 3 min and then the spores were incubated with BmE cells at 10:1 ratio in Grace’s medium at 28°C. The medium was replaced after 2 h, and the cells were cultured at 28°C in Grace’s medium supplemented with 10% (V/V) fetal bovine serum (FBS) (Gibco) and 1% (V/V) penicillin-streptomycin. Silkworms were raised to the third day of the fourth instar, and then were fed on mulberry leaves (1 cm^2^) spread with 10^5^ mature spores.

### Tissue paraffin section

For histological analysis of midgut cell apoptosis (infected and uninfected), the midgut of the fourth abdomen was isolated and fixed with 4% paraformaldehyde for 24 h at room temperature, then dehydrated through the series of ethanol, and embedded in paraffin. Then this material was sectioned into 5-μm slices and placed on slides. After deparaffinization and hydration, sections of the slides were stained with hoechst33342 and TUNEL to analyze the apoptosis of midgut cells.

### Terminal uridine nick-end labeling (TUNEL) assay

Apoptosis in Silkworm midgut and cultured BmE cells was detected using a terminal deoxynucleotidyl transferase-mediated dUTP-biotin nick end labeling (TUNEL) assay (Beyotime). For this assay, the midguts of infected silkworms were fixed in 4% paraformaldehyde, embedded in paraffin for 24 h, sectioned into 5-μm slices, and placed on slides. After deparaffinization and hydration, the slides were stained with TUNEL by incubation with TdT Reaction Buffer at 37°C for 10 min, followed by the incubation with a TdT reaction mixture at 37°C for 1 h. The slides were then incubated with a TUNEL reaction cocktail at 37°C for 30 min, counterstained with Hoechst33342. Images were acquired using a confocal microscope (OLYMPUS). For this assay, BmE cells were cultured in twelve well plates and infected with 1×10^6^ spores for 48 h. Cells were fixed with 4% paraformaldehyde for 15 min then incubated with PBS containing 0.3% Triton X-100 incubated for 5 min at room temperature. The cells were then stained with TUNEL and IFA. Images were acquired using a confocal microscope.

### Indirect Immunofluorescence Assay (IFA)

In order to characterize the location of NbSPN14 in the process of infection. BmE cells infected for varying lengths of time with *N*. *bombycis* were fixed with 4% paraformaldehyde for 10 min at room temperature, washed three times with 1×PBS, and then permeabilized using 0.1% Triton X-100 for 15 min. The cells were then blocked in 1×PBST containing 5% BSA and 10% goat serum for 1 h at room temperature. Next, the cells were incubated with mouse and rabbit polyclonal antibodies against NbSPN14 (anti-NbSPN14) and *N*. *bombycis* (anti-*N*. *b* total protein) diluted 1:100 in a blocking solution for 2 h at room temperature. The cells were then washed three times with 1xPBST, and incubated for 1 h with a 1:1000 dilution of Alexa Fluor 488 conjugate Goat anti-Mouse IgG (Invitrogen) and Alexa Fluor 594 conjugate Goat anti- Mouse IgG (Invitrogen) in a dark moist chamber at room temperature. The cell nucleus was stained with DAPI (1:1000 dilution, Sigma-Aldrich) at room temperature for 15 min. The samples were finally observed and photographed using an Olympus FV1200 laser scanning confocal microscope.

### Yeast signal sequence trap system

We used the yeast signal sequence trap system based on vector pSUC2T7M13ORI (pSUC2), which carries a truncated invertase gene, SUC2, lacking both the initiation Met and signal peptide [66]. DNA fragments coding for the signal peptides of NbSPN14 were synthesized by Sangon biotech and introduced into pSUC2 using Not I and Xho I restriction sites to create in-frame fusions to the invertase. Next, the invertase negative yeast strain YTK12 was transformed with 20 ng of each one of the pSUC2-derived plasmids individually using the lithium acetate method (we used plasmid PS87 as a positive control) [67]. After transformation, yeast was plated on CMD-W (minus Trp) plates (0.67% yeast N base without amino acids, 0.075% W dropout supplement, 2% sucrose, 0.1% glucose, and 2% agar). Transformed colonies were transferred to fresh CMD-W plates and incubated at 30°C, and transformation status was confirmed by PCR with vector-specific primers. To assay for invertase secretion, colonies were replica plated on YPRAA plates (1% yeast extract, 2% peptone, 2% raffinose) containing raffinose and lacking glucose. Also, invertase enzymatic activity was detected by the reduction of TTC to insoluble red colored triphenylformazan as follows. Five milliliters of CMD-W media (The YTK12 non-transfected strain was cultured in YPDA medium) were inoculated with the yeast transformants and incubated for 24 h at 30°C. Then, the pellet was collected, washed, and resuspended in distilled sterile water, and an aliquot was incubated at 35°C for 35 min with 0.1% of the colorless dye TTC. Colorimetric change was checked after 5 min incubation at room temperature.

### Protein structure prediction

To predict the structure of NbSPN14 using AlphaFold online website (https://alphafold.com/), first use its amino acid sequence and conduct a homology search to find related sequences. Next, generate a Multiple Sequence Alignment (MSA) to provide evolutionary context, and train AlphaFold’s neural network with this data. The network will then predict NbSPN14’s 3D structure, including secondary structures. Refine the model through molecular dynamics simulations, validate it using Ramachandran plots, and visualize the structure for detailed analysis [68]. Additionally, the structures of BmICE and the NbSPN14 RCL region P1 variant were predicted using AlphaFold. NbSPN14 and BmICE molecular docking was performed using AlphaFold 3 predictions (AlphaFold Server) [69]. Structure visualizations were created in PyMOL v.2.55.5 (Pymol GitHub Repository) [70].

### Western blot

Western blotting was used to detect the expression of NbSPN14 in infected cells, the total protein of BmE cells infected with *N*. *bombycis* was extracted using 300 μL RIAP strong lysis buffer containing PMSF ice for 15 min, and then centrifuged at 12,000 g at 4°C for 15 min. The supernatant was then used for 10% SDS-PAGE and then transferred to a PVDF membrane (Roche). The PVDF membrane was then blocked with 5% (w/v) skim milk (37°C, 1 h), followed by incubation with the antibody for 1.5 h at room temperature. Membranes were then washed 3 times with TBST buffer (10 mM Tris, 150 mM NaCl, 0.1% Tween-20), incubated with goat anti-mouse/rabbit IgG peroxidase antibody for 50 min at room temperature, and washed 3 times with TBST buffer. The antibody binding was detected using Clarit Western ECL substrate (Bio-Rad). Western blot experiments, such as detecting protein expression in transgenic cells and verifying interactions used the same methods as described above.

### Construction of NbSPN14 transgenic cell lines

The amino acid sequence of NbSPN14 was submitted to SignalP 6.0 Server (http://www.cbs.dtu.dk/services/SignalP/) and NCBI (https://www.ncbi.nlm.nih.gov/) for the signal peptide and domain predictions. The *NbSPN14* of *N*. *bombycis* (GenBank Accession No. FJ705061.1) was amplified from genomic DNA (gDNA) by PCR. The NbSPN14 signal peptide sequence was removed, and the forward primer containing a BamH I restriction site and the reverse primer containing a Not I restriction site were used. The amplification reaction consisted of 30 cycles of 95°C for 15 s, 55°C for 30 s, and 72°C for 1 min. The PCR products were recovered, integrated into the pSL1180 [IE2-SV40] vector, then the [IE2-NbSPN14-SV40] sequence fragment was amplified by primer pairs ‘pBac-F—pBac-R’ and ligated into the PiggyBac vector [A3-EGFP-SV40+A3-Neo-SV40-]. The recombinant vector PiggyBac [A3-EGFP-SV40+A3-Neo-SV40+IE2-NbSPN14-SV40] was extracted from the DH5α cells with the TIANpure Mini Plasmid Kit (TIANGEN). BmE cells were transfected with 2 μg of this recombinant plasmid and A3 helper plasmid using X-tremeGENE HP DNA Transfection Reagent (Roche), and the culture medium was changed after 6 h. Three days later, the cells were cultured in Grace insect complete medium containing G418 (200 μg/mL) (Merck), and the culture medium was changed once every 3 d. Screening continued for two months until the proportion of cells with fluorescent green exceeded 98% [71].

### CCK8 assay

The cell proliferation of BmE cells was tested using cell counting kit-8 (CCK8) (MCE, HY-K0301). According to the instructions of manufacturer, BmE cells in the logarithmic growth phase were seeded into the 96-well plates (5000 cells/well). Cells were incubated at 28°C for 0, 24, 48, 72, or 96 h and 10 μL of CCK8 reagent was added to each well After 3 h of incubation, the absorbance at 450 nm was measured to determine the cell viability using a multifunctional enzyme-labeling instrument. All experiments were performed in triplicate.

### Caspase 3/9 activity assay

NbSPN14 Transgenic cells were seeded into a 6-well culture plate and incubated in Grace supplemented with 10% FBS. After being treated as described above, protein extracts were prepared following the manufacturer’s instructions using a Bradford Protein Assay kit (Beyotime). Caspase 3 activity was measured using a Caspase 3 Activity Assay kit (Beyotime) in which cell extracts were mixed with Ac-DEVD-*p*NA substrate for 2 h at 37°C in 96-well plates prior to colorimetric measurement of *p*-nitroanilide product at 405 nm. The Caspase 9 activity assay was the same as the Caspase 3 activity assay, except that Ac-LEHD-*p*NA is used as the substrate.

### RNA interference

The sequence of *NbSPN14* was submitted to BLOCK-iT RNAi Designer (http://rnaidesigner.thermofisher.com/rnaiexpress/design.do). Two fragments that contain five potential interferential dsRNA fragments were amplified by the primers NbSPN14-1T7 and NbSPN14-2T7 (S1 Table). A DsRNA-EGFP fragment was used as the mock group [72]. The amplified product was used as a template to synthesize dsRNA using a RiboMAX Large Scale System-T7 Kit (Promega). The dsRNA was then isolated and purified, stored at -80°C. To evaluate the effect of RNAi, the ds-RNA and X-tremeGENE HP DNA Transfection Reagent (Roche) were mixed at a ratio of 1:1 (m/v) and added dropwise to the BmE cells, which were then infected with *N*. *bombycis*. Host cell apoptosis was analyzed at 1 d, 3 d and 5 d post infection.

To block the expression of NbSPN14 in silkworms, 10 μL (3 μg) dsRNA was injected into the silkworm hemocoel of the fifth instar; EGFP gene dsRNA was injected into the hemocoel of the control insects. These injections were performed immediately after the silkworms were orally inoculated with *N*. *bombycis* spores. A second injection of the same dose was administered after 24 h. On the 3rd and 4th days post-infection, the silkworms were dissected to determine the apoptosis situation in the midgut cells. To confirm the interference efficiency, NbSPN14 transcript and protein levels were determined by qRT-PCR and western blot.

### Yeast two-hybrid analysis

A yeast two-hybrid assay was conducted to explore the interaction between NbSPN14 and BmICE. NbSPN14 was cloned into the pGADT7 vector, while BmICE was cloned into the pGBKT7 vector. Competent yeast cells were transformed with these plasmids utilizing the Yeast Maker Yeast Transformation System 2 (Takara). The interactions were assessed in synthetic dropout medium lacking leucine, tryptophan, histidine, and adenine, supplemented with X-α-gal. The positive control consisted of the fusion strain with pGBKT7-53 and pGADT7-T, while the negative control was the fusion strain with pGBKT7-lam and pGADT7-T.

### Co-Immunoprecipitation (Co-IP)

To further verify the interactions between NbSPN14 and BmICE, Co-IP was performed we simultaneously expressed fusion proteins: V5-tagged NbSPN14 and HA-tagged BmICE in BmE cells. The cell total protein samples were immunoprecipitated using a HA antibody, and the detection was performed using the V5 antibody. The V5 antibody confirmed the specificity of the NbSPN14 signal in immunoprecipitates, reverse verification can also be used to detect BmICE specific bands in immunoprecipitate. Protein A + G agarose beads (Bio-Rad) were bound to V5 or HA antibody for 30 min, washed 3 times with 0.1% PBST, incubated with protein that had been extracted from NbSPN14-V5 and BmICE-HA recombinant protein co-expressed cells at 4°C 4 h, and then washed 3 times. The beads were then added (60 μL) to 1 × SDS PAGE loading buffer, the samples were boiled (10 min), and then used for western blotting.

### Construction BmICE knockout cell lines

sgRNA was designed based on the *BmICE* gene sequence and the CRISPRdirect online website (http://crispr.dbcls.jp/). Two sgRNA were designed in the fourth and fifth exon regions of the *BmICE* gene. Design primers based on the *BmICE* gene target sequence for expressing the sgRNA sequence fragment, then anneal the forward and reverse primers to form double-stranded DNA with sticky ends, which is connected to the sgRNA transcription vector pSL1180-U6-sgRNA containing the U6 promoter. 5’ end of the first gRNA target of the BmICE gene, and 3’ end downstream of the second gRNA target, 1000~1500bp were selected respectively as the homologous arm sequence of the donor. The 5’ donor fragment, [oPIE1-*GFP*:*BleoR*-Sv40] fluorescence reporter resistance gene fragment, and the 3’ donor fragment were ligated into a pESI vector using a Hieff Clone Plus Multi One Step Cloning Kit (Yeasen). BmE cells were inoculated into a 6-well cell culture plate. Then, the pSL1180-U6-sgRNA vector and pESI donor vector were co-transfected into these BmE cells, and 72 h after transfection, the screening medium containing 200 μg / mL Zeocin was replaced. The nutrient medium was replaced every 2 d until the green fluorescent cell’s ratio reached 98%.

### RNA extraction and qRT–PCR

The infected BmE cells and NbSPN14 transgenic cells’ total RNA was isolated using TRIzol regent (Invitrogen), and the mRNA in this preparation was then reverse transcribed into cDNA using the Hifair AdvanceFast One-step RT-gDNA Digestion SuperMix for qPCR (Yeasen) according to the manufacturer’s instructions. qRT–PCR was then performed using the Hieff qPCR SYBR Green Master Mix (Yeasen), and *Nb-β tubulin* and *BmRPL3* were used as the internal control. Three biological replicates were performed for each experiment. All PCR primers used are listed in supplemental S1 Table.

### Statistics

The difference between control and experimental assays was evaluated using a two-tailed Student’s T test and single-factor ANOVA employing GraphPad Prism 5 software. Statistical differences between two groups of p < 0.001 (***) were extremely significant differences; p < 0.01 (**) were selected to indicate highly significant differences, while p < 0.05 (*) represented significant differences, and p ≥ 0.05 (NS) indicated the lack of significant difference.

## Supporting information

S1 Fig Observation of *N*. *bombycis* in the midgut of silkworms and Analysis of Caspase 3 activity after *N*. *bombycis* infection. A. Observation on the sections of silkworm midgut infected with *N*. *bombycis*, the white triangle is the *N*. *bombycis* nuclei marked by Hoechst33342, bar, 50 μm. B. The Caspase 3 activity of BmE cells infected with *N*. *bombycis* for 48 hours and treatment with Act D for 6 hours.
(TIF)

S2 Fig Transcriptional characterization of NbSPN14 in infected cell and expression localization of NbSPN14 in BmE cell. A. NbSPN14 Signal Peptide(1-19Aa), Serpin Domain and the predicted structure with Reactive Center Loop exposure, the typical structural characteristics of the serpin protein by AlphaFold Protein Structure Database. B. The protein expression level of NbSPN14 in infected BmE cell lines was analyzed by Western blot, the arrow refers to the NbSPN14 band. C. Analysis of NbSPN14 transcripts at 0, 2, 6, 12, 24, 36, 48, 72, and 96 h after infection with BmE cells showed that all transcripts were recorded, the highest level was at 48 h, then followed by 96 h after infection. D. The localization of NbSPN14 fusion protein was analyzed by IFA at 48 h after transfection of the psL1180-IE2-NbSPN14-V5 expression vector into BmE cells. The primary antibody uses the V5-tagged mouse monoclonal antibody, the secondary antibody was the Goat anti-Mouse IgG with Alexa Fluor 488. The control group expresses the EGFP protein fused with a V5 tag, which was directly observed under a confocal microscope in the Alexa 488 channel.
(TIF)

S3 Fig Inhibition of host cell apoptosis by overexpression of NbSPN14 in BmE cell line. A. Hochest33342 and Annexin V staining for apoptosis detection in live NbSPN14 transgenic cells. B. The quantitative analysis of the proportion of red fluorescence signal-positive cells out of the total cell population (n = 10).
(TIF)

S4 Fig *In vitro* and *in vivo* interference of NbSPN14 expression. A. At the cellular level, transcription analysis of NbSPN14 was performed following transfection with double-stranded fragments that interfered with NbSPN14, at 1-, 3-, and 5-days post infection. Transfections with EGFP interference fragments served as a control. B. Western blot was used to analyze the expression of NbSPN14 after transfection of interference fragments, and the transfected EGFP interference fragments were used as control. C. Quantitative analysis was conducted on the protein expression of NbSPN14 after interference. The relative expression level of NbSPN14 is the ratio of the gray level of the NbSPN14 band to that of the internal reference β-tubulin band. D. At the individual level, the transcription of NbSPN14 in the midgut was analyzed 3–4 days after injection of interference fragments into the stomata of silkworms infected with *N*. *bombycis*. E. Western blot was used to analyze the expression of NbSPN14 after transfection of interference fragments, and the transfected EGFP interference fragments were used as control. F. Quantitative analysis was conducted on the protein expression of NbSPN14 after interference. The relative expression level of NbSPN14 is the ratio of the gray level of the NbSPN14 band to that of the internal reference β-tubulin band.
(TIF)

S5 Fig Analysis of NbSPN14 inhibition of apoptosis pathway in the host cell. A. NbSPN14 transgenic cells inhibit host cell Caspase 3 activity induced by Ultraviolet, Staurosporine (STS), and Actinomycin D (Act D) treatment. Ac-DEVD-CHO is a Caspase 3 specific inhibitor, as positive control. B. The BmICE protein domain shows that BmICE has a pro domain and P10 and P20 subunits. C. Caspase 9 activity analysis of NbSPN14 transgenic cells treated with Act D.
(TIF)

S6 Fig BmICE plays an important role in silkworm apoptosis. A. The PIZT-[IE1-BmICE-HA-sv40] expression vector was constructed and transfected into BmE cells. BmICE-HA is able to localize to the host cytoplasm and nucleus. HA-tagged rabbit antibody was used to indicate the subcellular localization of BmICE-HA recombinant proteins. The Alexa594-conjugated goat anti-rabbit antibody is the secondary antibody. B. RT-qPCR analyzed the expression of *BmICE* in knockout cells. C. Western blot analyzed the expression of BmICE in knockout cells. D. In *BmICE* knockout cells, RT-qPCR analysis of the *BmBuffy*/*BmApaf1*/*BmDronc*/*BmDredd*/*BmICE* gene expression in the apoptosis pathway showed that there was no significant difference between the experimental and the control group, only the expression of BmICE was significantly down-regulated. E. After the *BmICE* knockout cells were treated with Act D for 12 hours, the apoptosis-related genes *BmBuffy*/*BmApaf1*/*BmDronc*/*BmDredd*/*BmICE* genes expression was analyzed by quantitative PCR. The results showed that the apoptosis pathway was activated, but BmICE was still at a low transcriptional level.
(TIF)

S1 Table List of primers used in this study. (DOC)

S2 Table Source data for figures. (XLSX)
