# Supplementary figures and images for: Microsporidian Nosema bombycis secretes serine protease inhibitor to suppress host cell apoptosis via Caspase BmICE
Source: PLoS Pathog. 2025 Jan 7;21(1):e1012373. doi: 10.1371/journal.ppat.1012373 (PMC11741654; doi:10.1371/journal.ppat.1012373)

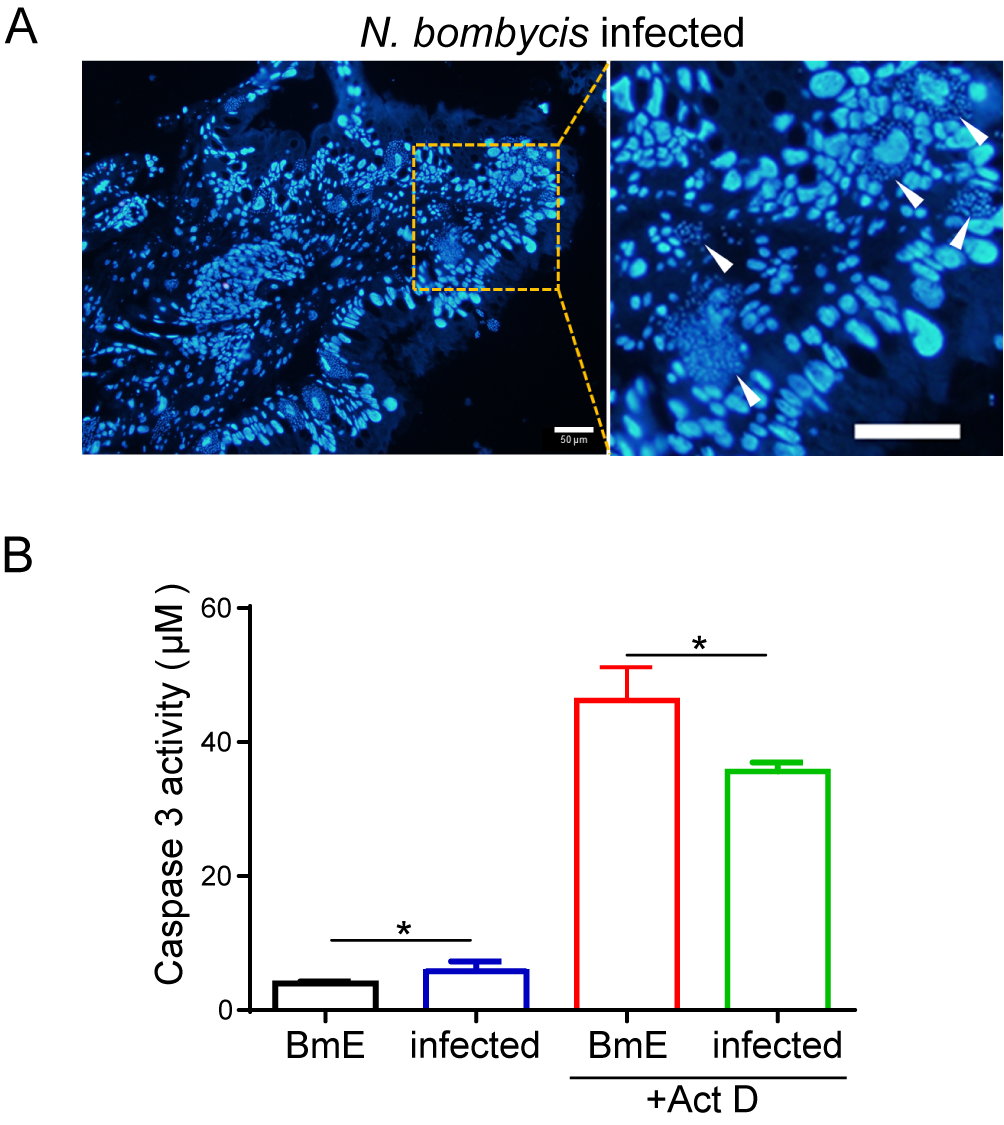

Supplement: S1 Fig — A. Observation on the sections of silkworm midgut infected with N. bombycis, the white triangle is the N. bombycis nuclei marked by Hoechst33342, bar, 50 μm. B. The Caspase 3 activity of BmE cells infected with N. bombycis for 48 hours and treatment with Act D for 6 hours. (TIF) [file ppat.1012373.s001.tif]

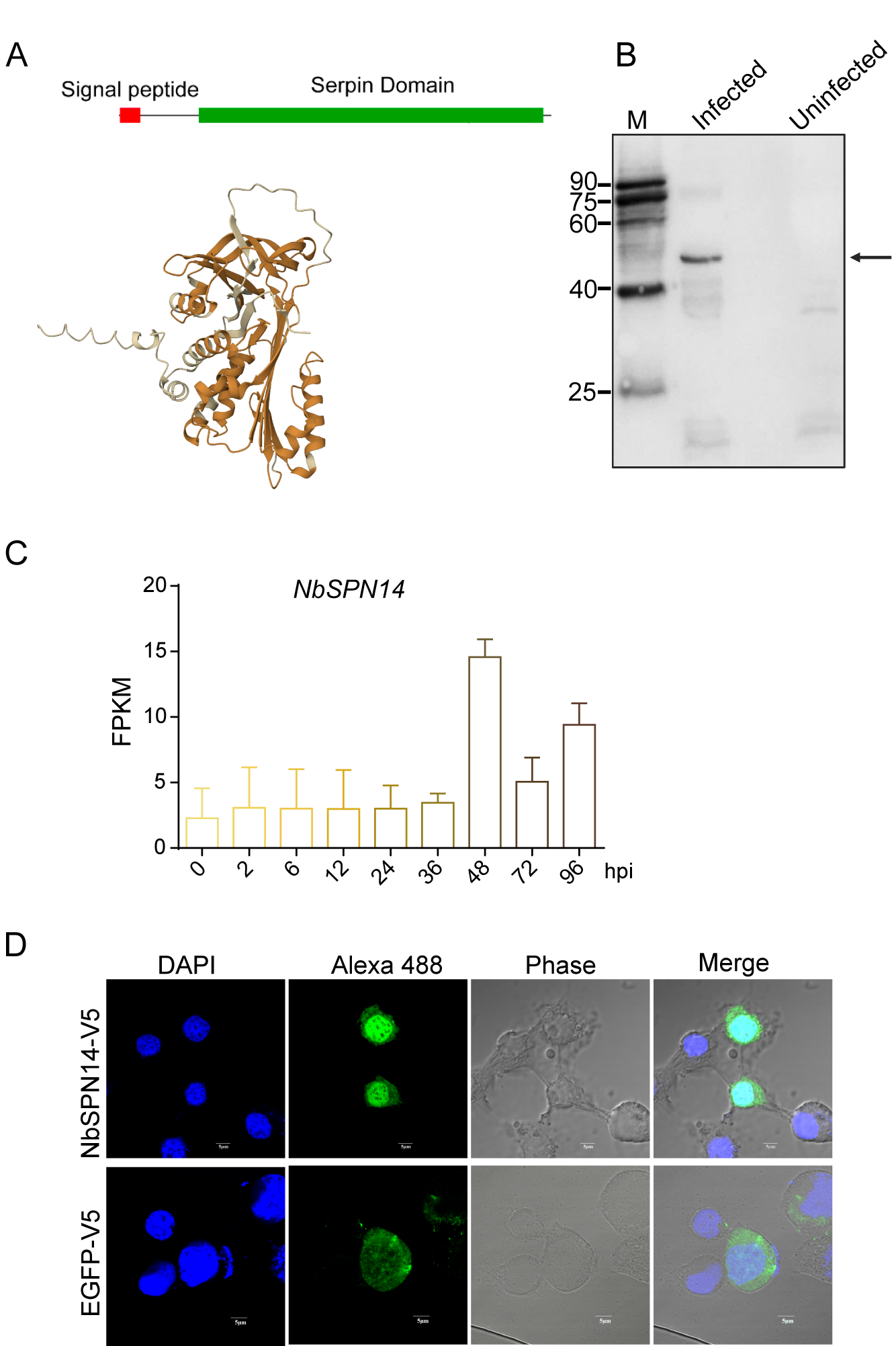

Supplement: S2 Fig — A. NbSPN14 Signal Peptide(1-19Aa), Serpin Domain and the predicted structure with Reactive Center Loop exposure, the typical structural characteristics of the serpin protein by AlphaFold Protein Structure Database. B. The protein expression level of NbSPN14 in infected BmE cell lines was analyzed by Western blot, the arrow refers to the NbSPN14 band. C. Analysis of NbSPN14 transcripts at 0, 2, 6, 12, 24, 36, 48, 72, and 96 h after infection with BmE cells showed that all transcripts were recorded, the highest level was at 48 h, then followed by 96 h after infection. D. The localization of NbSPN14 fusion protein was analyzed by IFA at 48 h after transfection of the psL1180-IE2-NbSPN14-V5 expression vector into BmE cells. The primary antibody uses the V5-tagged mouse monoclonal antibody, the secondary antibody was the Goat anti-Mouse IgG with Alexa Fluor 488. The control group expresses the EGFP protein fused with a V5 tag, which was directly observed under a confocal microscope in the Alexa 488 channel. (TIF) [file ppat.1012373.s002.tif]

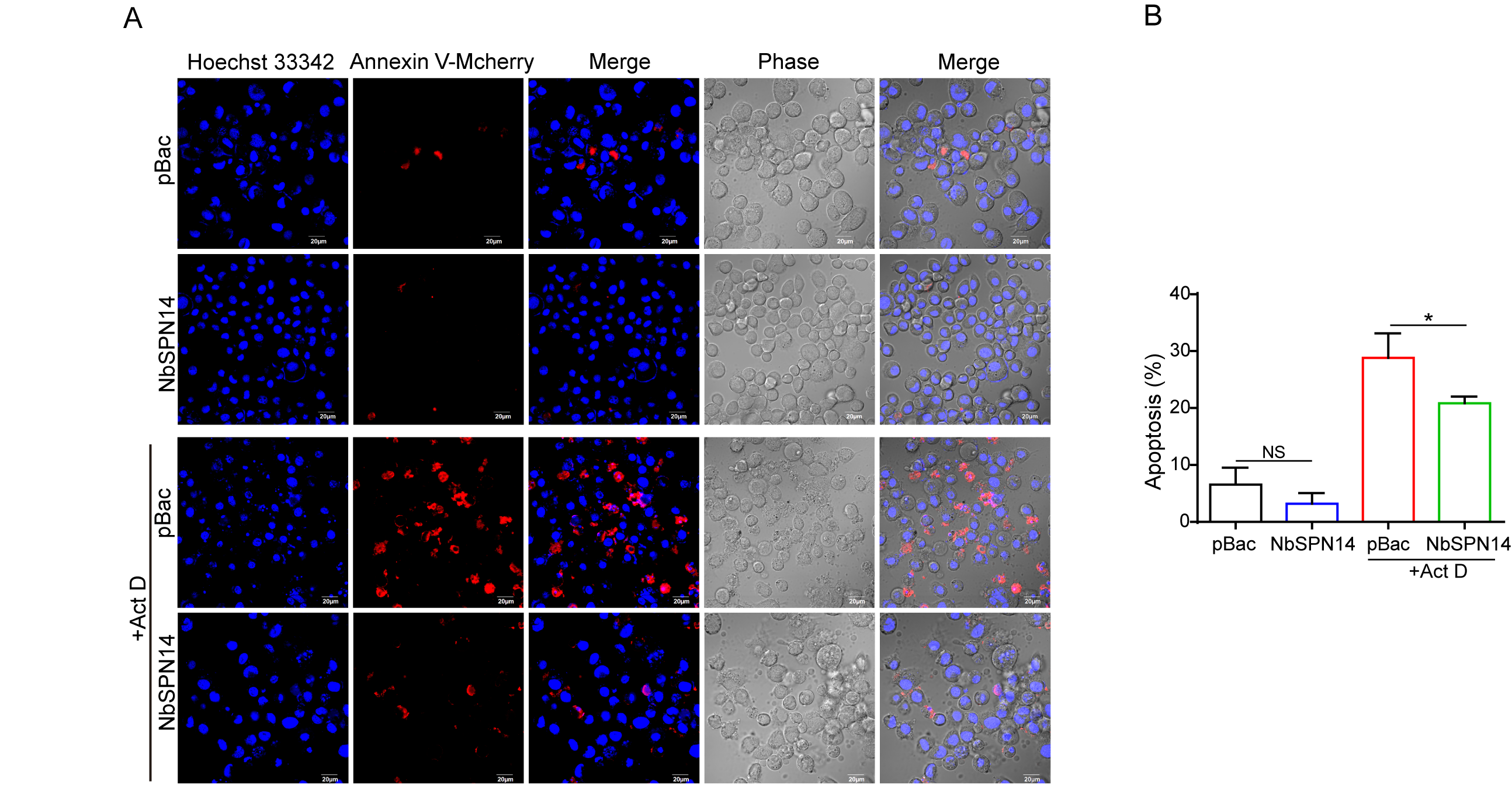

Supplement: S3 Fig — A. Hochest33342 and Annexin V staining for apoptosis detection in live NbSPN14 transgenic cells. B. The quantitative analysis of the proportion of red fluorescence signal-positive cells out of the total cell population (n = 10). (TIF) [file ppat.1012373.s003.tif]

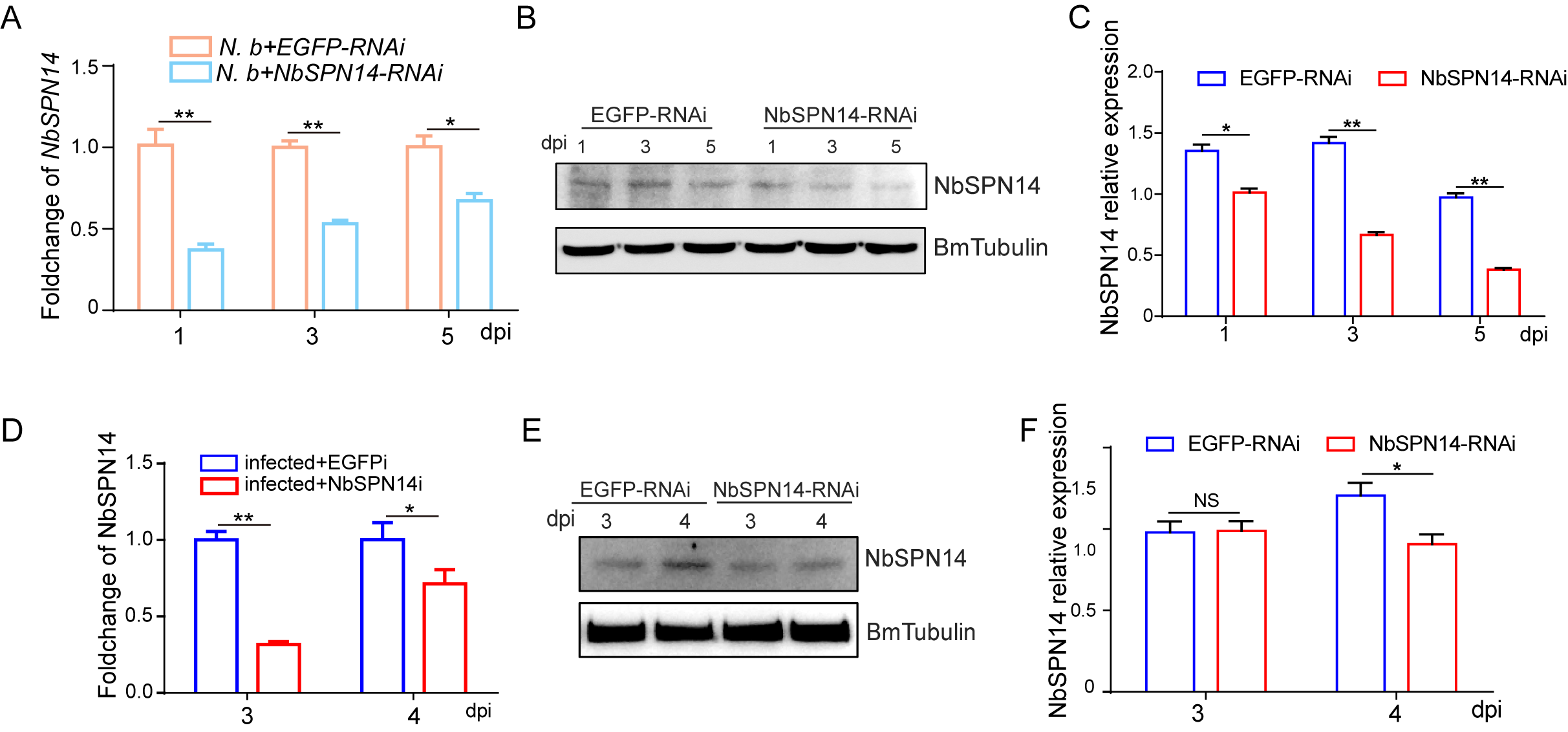

Supplement: S4 Fig — A. At the cellular level, transcription analysis of NbSPN14 was performed following transfection with double-stranded fragments that interfered with NbSPN14, at 1-, 3-, and 5-days post infection. Transfections with EGFP interference fragments served as a control. B. Western blot was used to analyze the expression of NbSPN14 after transfection of interference fragments, and the transfected EGFP interference fragments were used as control. C. Quantitative analysis was conducted on the protein expression of NbSPN14 after interference. The relative expression level of NbSPN14 is the ratio of the gray level of the NbSPN14 band to that of the internal reference β-tubulin band. D. At the individual level, the transcription of NbSPN14 in the midgut was analyzed 3–4 days after injection of interference fragments into the stomata of silkworms infected with N. bombycis. E. Western blot was used to analyze the expression of NbSPN14 after transfection of interference fragments, and the transfected EGFP interference fragments were used as control. F. Quantitative analysis was conducted on the protein expression of NbSPN14 after interference. The relative expression level of NbSPN14 is the ratio of the gray level of the NbSPN14 band to that of the internal reference β-tubulin band. (TIF) [file ppat.1012373.s004.tif]

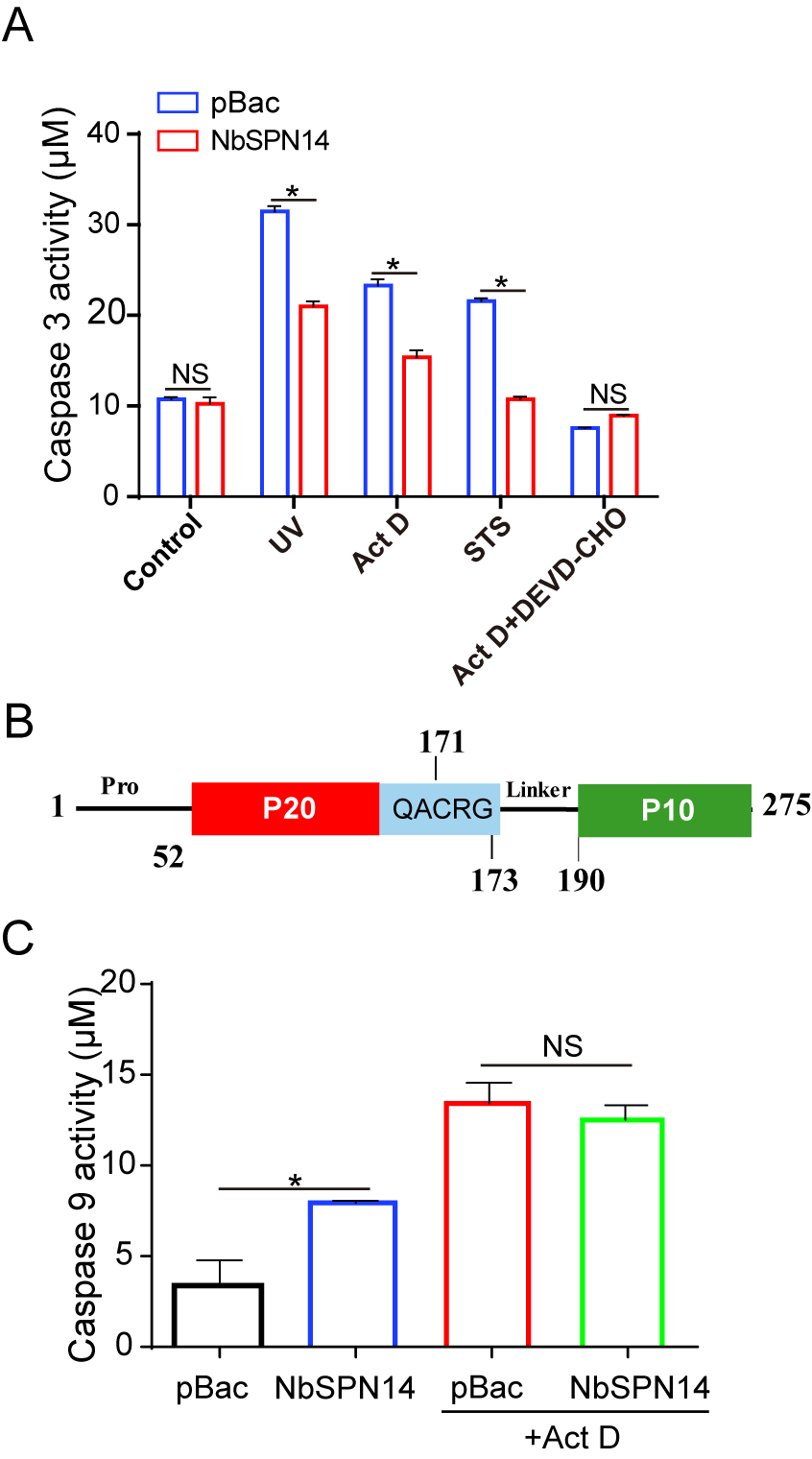

Supplement: S5 Fig — A. NbSPN14 transgenic cells inhibit host cell Caspase 3 activity induced by Ultraviolet, Staurosporine (STS), and Actinomycin D (Act D) treatment. Ac-DEVD-CHO is a Caspase 3 specific inhibitor, as positive control. B. The BmICE protein domain shows that BmICE has a pro domain and P10 and P20 subunits. C. Caspase 9 activity analysis of NbSPN14 transgenic cells treated with Act D. (TIF) [file ppat.1012373.s005.tif]

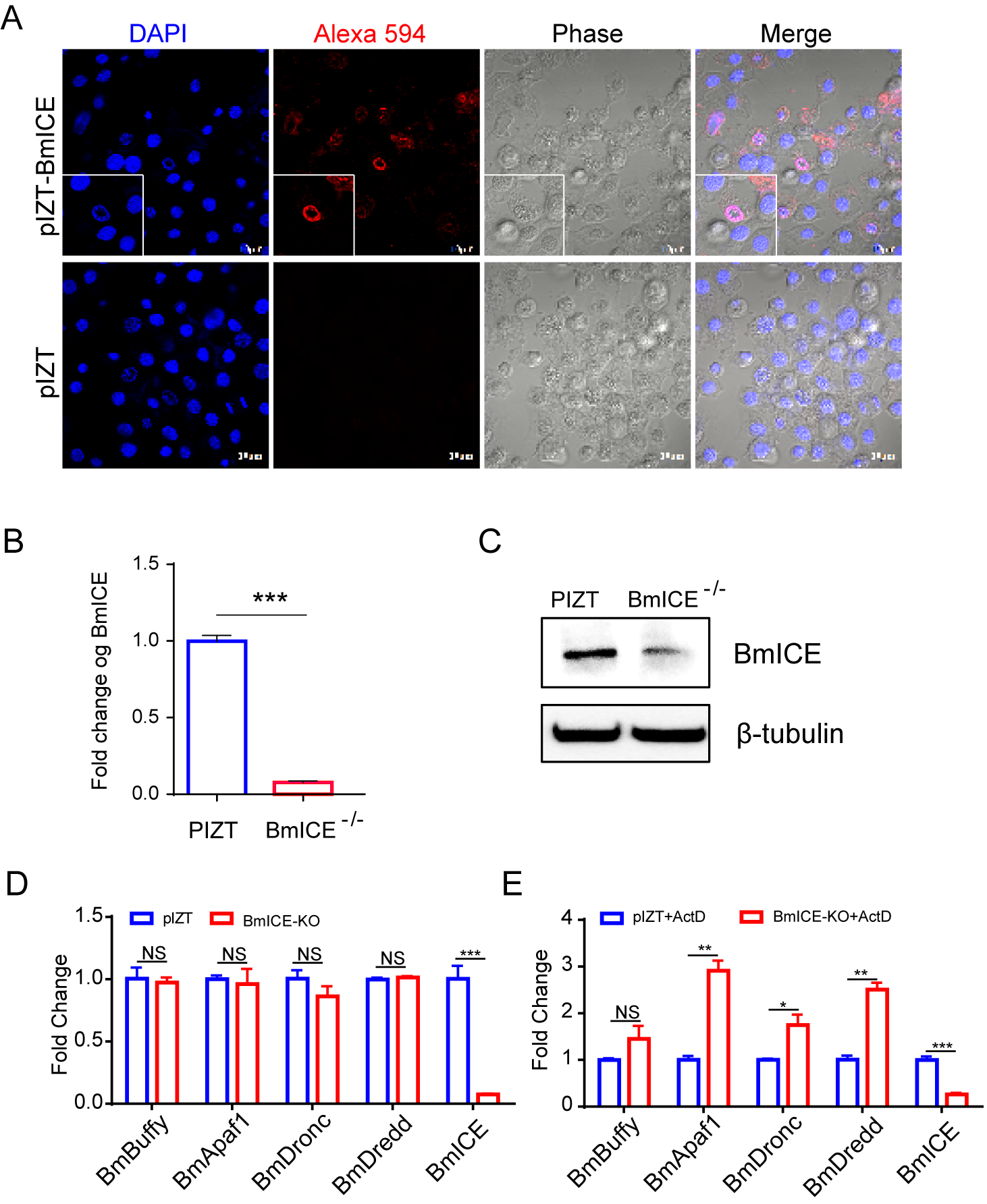

Supplement: S6 Fig — A. The PIZT-[IE1-BmICE-HA-sv40] expression vector was constructed and transfected into BmE cells. BmICE-HA is able to localize to the host cytoplasm and nucleus. HA-tagged rabbit antibody was used to indicate the subcellular localization of BmICE-HA recombinant proteins. The Alexa594-conjugated goat anti-rabbit antibody is the secondary antibody. B. RT-qPCR analyzed the expression of BmICE in knockout cells. C. Western blot analyzed the expression of BmICE in knockout cells. D. In BmICE knockout cells, RT-qPCR analysis of the BmBuffy/BmApaf1/BmDronc/BmDredd/BmICE gene expression in the apoptosis pathway showed that there was no significant difference between the experimental and the control group, only the expression of BmICE was significantly down-regulated. E. After the BmICE knockout cells were treated with Act D for 12 hours, the apoptosis-related genes BmBuffy/BmApaf1/BmDronc/BmDredd/BmICE genes expression was analyzed by quantitative PCR. The results showed that the apoptosis pathway was activated, but BmICE was still at a low transcriptional level. (TIF) [file ppat.1012373.s006.tif]
