# Supplementary material for: Microsporidian Nosema bombycis secretes serine protease inhibitor to suppress host cell apoptosis via Caspase BmICE
Source: PLoS Pathog. 2025 Jan 7;21(1):e1012373. doi: 10.1371/journal.ppat.1012373 (PMC11741654; doi:10.1371/journal.ppat.1012373)
Supplement: S1 Table — (DOC) [file ppat.1012373.s007.doc]

**S1 Table. List of primers used in this study**

| **Primers** | **Sequences of oligonucleotides（5’→3’）** | **Function** |
| --- | --- | --- |
| NbSPN14-F | CGACTAGGGATCTAGGATCCATGTTACCTGATGCAAAAAATGATT | [Clone](https://cn.bing.com/dict/search?q=Cloning&FORM=BDVSP6&cc=cn) and [expression](https://cn.bing.com/dict/search?q=expression&FORM=BDVSP6&cc=cn) |
| NbSPN14-R | ACATCGTATGGGTACCCGGGTTCTTTAATTATTCCAGTGTCTTTG |
| BmICE-HA-F | GTTCGAATTTAAAGCTTGGTACCATGGAGGAGACTTCGAACGATT | [Clone](https://cn.bing.com/dict/search?q=Cloning&FORM=BDVSP6&cc=cn) and [expression](https://cn.bing.com/dict/search?q=expression&FORM=BDVSP6&cc=cn) |
| BmICE-HA-R | GCCCTCTAGACTCGAGCGGCCGCTTACTCGAGAGCGTAATCTGGA |
| pBac-F | CCGGGTACCGTCGACGGCGCGCCCATGATGATAAACAATGTATGGTGC | Transgenic vector construction |
| pBac-R | CATTGTTTATCATCATGGCGCGCCCACGCGCTTGAAAGGAGTGTGTAA |
| NbSPN14-DA-F | GAAATATGTTGCCTGTTGCCATATAACAGTTAAAGCTG | Site-directed mutagenesis |
| NbSPN14-DA-R | ATGGCAACAGGCAACATATTTCGATAATAAGCAAATAAC |
| NbSPN14-AAA-F | ATCGAAATATGCTGCCGCTTGCCATATAACAGTTAAAGCTGATAG |
| NbSPN14-AAA-R | GTTATATGGCAAGCGGCAGCATATTTCGATAATAAGCAAATAAC |
| NbSPN14-1T7-F | TAATACGACTCACTATAGGGACATTGAATTGTTTATACGAA | *NbSPN14* RNAi |
| NbSPN14-1T7-R | TAATACGACTCACTATAGGGTTGAAGTAAACCAAAGAAGC |
| NbSPN14-2T7-F | TAATACGACTCACTATAGGGTGGGACGCATTCCGAGCATCT |
| NbSPN14-2T7-R | TAATACGACTCACTATAGGGGCTTTAACTGTTATATGGCAA |
| sgRNA-1-F | AAGTGGTGCTGGATGGTGAAAGCA | *BmICE* knock out |
| sgRNA-1-R | AAACTGCTTTCACCATCCAGCACC |
| sgRNA-2-F | AAGTGATCGTCACAATAACACATG |
| sgRNA-2-R | AAACCATGTGTTATTGTGACGATC |
| pESI- Doner1-F | CGTATTCGCGTGTCGCCCTTGGCGCGCCGTGTTTTAAGGTGAGTGGCGC |
| IE1-Doner1-R | TGCGCGCGCCGTGCGTGTTTCGCTAGCACGGTAAGAAGTAGAT |
| SV40-ICE Doner2-F | CCAAACTCATCAATGTATCTTACGTGTTCCAGTGCTTAGTAC |
| pESI-ICE Doner2-R | CGACTTCGCGTGTCGCCCTTGGCGCGCCCAGCGATGCCAGGAGCAAAG |
| BmICE-NdeI-F | GTACCAGATTACGCTCATATGATGGAGGAGACTTCGAACGATT | Yeast two-hybrid |
| BmICE-BamHI-R | CAGCTCGAGCTCGATGGATCCCTACTTTTCAAACATAAATAAC |
| NbSPN14-NdeI-F | GTACCAGATTACGCTCATATGTTACCTGATGCAAAAAATGATTC |
| NbSPN14-BamHI-R | CAGCTCGAGCTCGATGGATCCTTATTCTTTAATTATTCCAGTGTC |
| NbSPN14-qF | GACGCATTCCGAGCATCTT | Quantitative PCR |
| NbSPN14-qR | AATCCCGCAGAATCAAAGG |
| NbTubulin-qF | AGAACCAGGAACAATGGACG |
| NbTubulin-qR | AGCCCAATTATTACCAGCACC |
| BmRPL3-qF | CGGTGTTGTTGGATACATTGAG |
| BmRPL3-qR | GCTCATCCTGCCATTTCTTACT |
| EGFP-qF | CTGACCCTGAAGTTCATCTGCA |
| EGFP-qR | AAGAAGATGGTGCGCTCCTG |
| BmDredd-qF | TAATAGTCGTTCTGACTTGGGACA |
| BmDredd-qR | TCGGTATGCAATGCAGTTTCT |
| BmApaf1-qF | TATGCTGCGTCCCCTG |
| BmApaf1-qR | GTGCCATTATCTCGTTTTG |
| Bmbuffy-qF | GCTATGTGCGGCGTTGGAG |
| Bmbuffy-qR | CCCTTGTGACCCGTCTTGC |
| BmCytc-qF | TCATACTCCGATGCCAATA |
| BmCytc-qR | TAGGCAATAAGGTCAGCAC |
| BmDronc-qF | CGAGTGCGTGTTCCTGGTGGTGT |
| BmDronc-qR | CTGGGAGGGTGGAGTTAGCGATG |
| BmICE-qF | TCTGTTGACGGTTATCTTTC |
| BmICE-qR | TATTGTTGGTCTCCTGACAT |
